# Self‐Adaptive Allantoin@ZIF8 Nanocomposite Hydrogel with Resveratrol Synergy for MRSA‐Infected Wound Regeneration

**DOI:** 10.1002/advs.202520614

**Published:** 2026-03-16

**Authors:** Yongjie Zhu, Dun Liu, Mengran Zhao, Xinyi Jian, Enuo Peng, Xingping Zhao, Zezhang Zhu, Bolin Tang, Benlong Shi

**Affiliations:** ^1^ Division of Spine Surgery Department of Orthopedic Surgery Nanjing Drum Tower Hospital Affiliated Hospital of Medical School Nanjing University Nanjing China; ^2^ School of Materials and Textile Engineering Jiaxing University Jiaxing China; ^3^ Department of Gynecology Third Xiangya Hospital Central South University Changsha China; ^4^ Nanotechnology Research Institute G60 STI Valley Industry & Innovation Institute Jiaxing University Jiaxing China; ^5^ Zhejiang Key Laboratory of Bio‐based Health Functional Fiber Materials Jiaxing University Jiaxing China

**Keywords:** antibacterial activity, allantoin, resveratrol, wound healing, zeolitic imidazolate framework 8

## Abstract

Infected wounds present a formidable challenge to healing due to their complex pathological microenvironment, whereas conventional dressings are often functionally limited and ill‐suited to adapt to dynamic wound changes. In this study, based on allantoin‐loaded ZIF8 nanoparticles (Alla@ZIF8) and methacrylated quaternary ammonium carboxymethyl chitosan/resveratrol composite hydrogels (RMQCC), we designed and developed an adaptive multifunctional hydrogel system (Alla@ZIF8‐Gel). This nanocomposite hydrogel exhibited multiple intelligent features, including pH‐responsiveness and morphological adaptability. The quaternary ammonium carboxymethyl chitosan imparted well biocompatibility and robust antimicrobial properties. Electrostatic interactions and hydrogen bonds endowed hydrogels with excellent injectability and self‐healing capabilities. The ZIF8 nanoparticles enabled the intelligent, on‐demand release of allantoin, thereby promoting tissue regeneration. Resveratrol conferred potent antioxidant, anti‐inflammatory, and immunomodulatory effects. The Alla@ZIF8‐Gel dynamically modulated drug release kinetics in response to the wound microenvironment, synergistically delivering antibacterial, antioxidant, anti‐inflammatory, and tissue‐regenerative functions. Both in vitro and in vivo experimental results demonstrated that this adaptive hydrogel markedly accelerated the healing of MRSA‐infected wounds, suppressed bacterial proliferation, alleviated oxidative damage and inflammatory responses, and enhanced collagen deposition, re‐epithelialization, and neovascularization. This work provides a novel design paradigm for the development of intelligent, multifunctional therapeutic materials for infected wound management, with significant translational potential for clinical application.

## Graphical abstract

Based on allantoin‐loaded ZIF8 nanoparticles (Alla@ZIF8) and methacrylated quaternary ammonium carboxymethyl chitosan/resveratrol composite hydrogels (RMQCC), an adaptive multifunctional hydrogel system (Alla@ZIF8‐Gel) was constructed and confirmed to induce the healing of MRSA‐infected wounds via suppressing bacterial proliferation, alleviating oxidative damage and inflammatory responses, and enhancing collagen deposition, re‐epithelialization, and neovascularization.

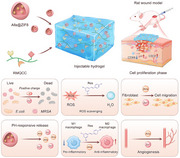

## 1 Introduction

Wound repair stands as a pivotal and enduring challenge in biomedical research, with the management of infected wounds presenting particularly formidable obstacles [1]. Traditional wound dressings typically exhibit limited functionality, rendering them inadequate for simultaneously addressing the multifaceted requirements of antimicrobial activity, anti‐inflammatory effects, promotion of angiogenesis, and tissue regeneration [2, 3, 4, 5]. Infected wounds are frequently accompanied by persistent oxidative stress, excessive inflammatory responses, and the formation of bacterial biofilms [6, 7]. These elements interact synergistically to create a complex pathological microenvironment that severely impedes normal wound healing processes. Although a variety of antimicrobial agents and growth factors have been employed in wound treatment, their clinical efficacy often remains suboptimal due to challenges such as maintaining effective local concentrations, susceptibility to washout by wound exudate, and the potential development of microbial resistance [8]. Consequently, the development of multifunctional biomaterials capable of concurrently countering infection, oxidative stress, and inflammation, while enabling sustained release of bioactive molecules, is of profound significance for enhancing therapeutic outcomes in infected wound treatment [9]. Of particular significance is the dynamic nature of the wound microenvironment throughout the healing process. Conventional static dressings are inherently ill equipped to adapt to these evolving conditions [10, 11, 12]. In hence, the development of hydrogel systems with adaptive capabilities, capable of dynamically modulating their functions in response to the distinct pathophysiological features characteristic of different wound stages, has emerged as a pivotal research direction in the field of advanced wound repair materials.

Carboxymethyl chitosan (CC), a natural polysaccharide derivative, has emerged as a highly promising candidate for wound repair applications, owing to its excellent biocompatibility, biodegradability, non‐toxicity, and inherent bioactivity [13, 14]. In contrast to native chitosan, CC exhibits markedly enhanced water solubility, facilitating its processing into diverse forms of biomaterials [15]. Furthermore, it demonstrates pro‐healing properties, including the promotion of fibroblast proliferation, acceleration of collagen deposition, and enhancement of epithelialization, rendering it an ideal substrate for wound dressings. Nevertheless, the clinical translation of native carboxymethyl chitosan is constrained by its limited intrinsic antimicrobial activity, suboptimal mechanical properties, and a lack of responsiveness to the complex wound microenvironment. Previous research has predominantly focused on single‐function modifications, such as solely enhancing antimicrobial efficacy or improving mechanical strength, which fail to meet the multifaceted requirements for treating infected wounds [16, 17]. In this study, we report a dual‐functionalization strategy involving methacrylation and quaternization of carboxymethyl chitosan (MQCC). This approach not only substantially augments its antimicrobial potency but also confers injectability and photocurable properties, enabling the material to conform to irregular wound geometries and form a stable protective barrier. This synergistic, multifunctional modification strategy represents a significant advancement over the single‐function approaches documented in prior literature. Moreover, in this study, the introduction of quaternary ammonium groups into carboxymethyl chitosan not only markedly enhanced its antimicrobial efficacy but also endowed the material with cation‐responsive properties. This enables the hydrogel to modulate its swelling behavior and drug release kinetics in response to fluctuations in pH and ionic strength within the wound microenvironment. Such adaptive characteristics are particularly crucial for navigating the dynamically evolving pathophysiological landscape of healing wounds.

Zeolitic imidazolate framework 8 (ZIF8), a metal–organic framework constructed from zinc ions (Zn^2^^+^) and imidazolate linkers, is distinguished by its high porosity, tunable surface chemistry, and excellent biocompatibility [18, 19]. Furthermore, ZIF8 exhibits considerable promise in the field of drug delivery owing to its unique pH‐responsive degradation profile [20]. While remaining stable under physiological conditions, ZIF8 undergoes rapid degradation in the acidic microenvironment of infected wounds (pH 5.5∼6.5), thereby facilitating the release of encapsulated therapeutic molecules. This intelligent responsiveness renders ZIF8 an ideal candidate for microenvironment‐adaptive drug delivery in wound healing applications. Additionally, the sustained release of zinc ions endows ZIF8 with inherent antimicrobial properties, further underscoring its prominence in advanced drug delivery systems. In contrast to conventional inorganic nanocarriers, ZIF8 offers superior drug‐loading capacity and more precisely controlled release kinetics, enabling the maintenance of effective local drug concentrations and prolonging therapeutic action. Allantoin (Alla), a naturally derived bioactive compound, plays a pivotal role in wound repair by virtue of its multifaceted biological functions, including promotion of cell proliferation, acceleration of epithelialization, anti‐inflammatory activity, and moisturization [21]. However, its poor water solubility and rapid clearance by wound exudate limit its sustained efficacy upon topical administration. In this study, by encapsulating Alla within ZIF8 nanoparticles, we not only circumvented the limitations of solubility and stability but also achieved localized sustained release, thereby significantly extending its duration of action. Compared with previously reported methods involving the direct physical blending of Alla into hydrogels, this ZIF8‐mediated delivery system more effectively preserves the bioactivity of allantoin and affords finer control over its release profile. Consequently, it enables more precise and sustained modulation throughout the various stages of wound repair, representing a substantial improvement over the conventional physical mixing strategy. Most importantly, the selection of ZIF8 as an Alla carrier in this work capitalizes not only on its exceptional drug‐loading and protective capabilities but, more critically, on its pH‐responsive release characteristics. This facilitates accelerated release of Alla in response to increased acidity within infected regions, while slowing release as inflammation resolves and pH returns to normal. As a result, a smart drug release modality is achieved that is temporally synchronized with the wound healing trajectory, offering a sophisticated and adaptive therapeutic paradigm.

Oxidative stress represents a pivotal impediment to the healing of infected wounds, wherein excessive reactive oxygen species (ROS) not only inflict direct cellular and tissue damage but also exacerbate inflammatory responses and impede angiogenesis, thereby delaying wound repair [22, 23, 24, 25]. Resveratrol (Res), a naturally occurring polyphenolic compound, exhibits potent antioxidant capacity, effectively scavenging diverse ROS, modulating redox homeostasis, and protecting cells from oxidative injury [26, 27]. In contrast to synthetic antioxidants, resveratrol offers superior biocompatibility and a broader spectrum of biological activities, including not only antioxidation but also anti‐inflammatory and pro‐angiogenic functions. Nevertheless, the clinical translation of Res has been markedly constrained by its poor water solubility, low photostability, and limited bioavailability. Previous studies have predominantly incorporated Res into hydrogels via simple physical blending, an approach that fails to resolve its inherent instability and may result in burst release, thereby compromising its long‐term therapeutic efficacy. In this study, Res was chemically conjugated into a quaternary ammonium carboxymethyl chitosan hydrogel network. This strategy not only enhanced the stability and dispersibility of Res but also facilitated its sustained local release at the wound site, effectively mitigating burst release phenomena. Compared with the physical mixing methods documented in the literature, this chemical conjugation approach more effectively preserves the bioactivity of Res and prolongs its duration of action, thereby enabling continuous antioxidant and anti‐inflammatory effects throughout the entire wound healing process.

**SCHEME 1:**
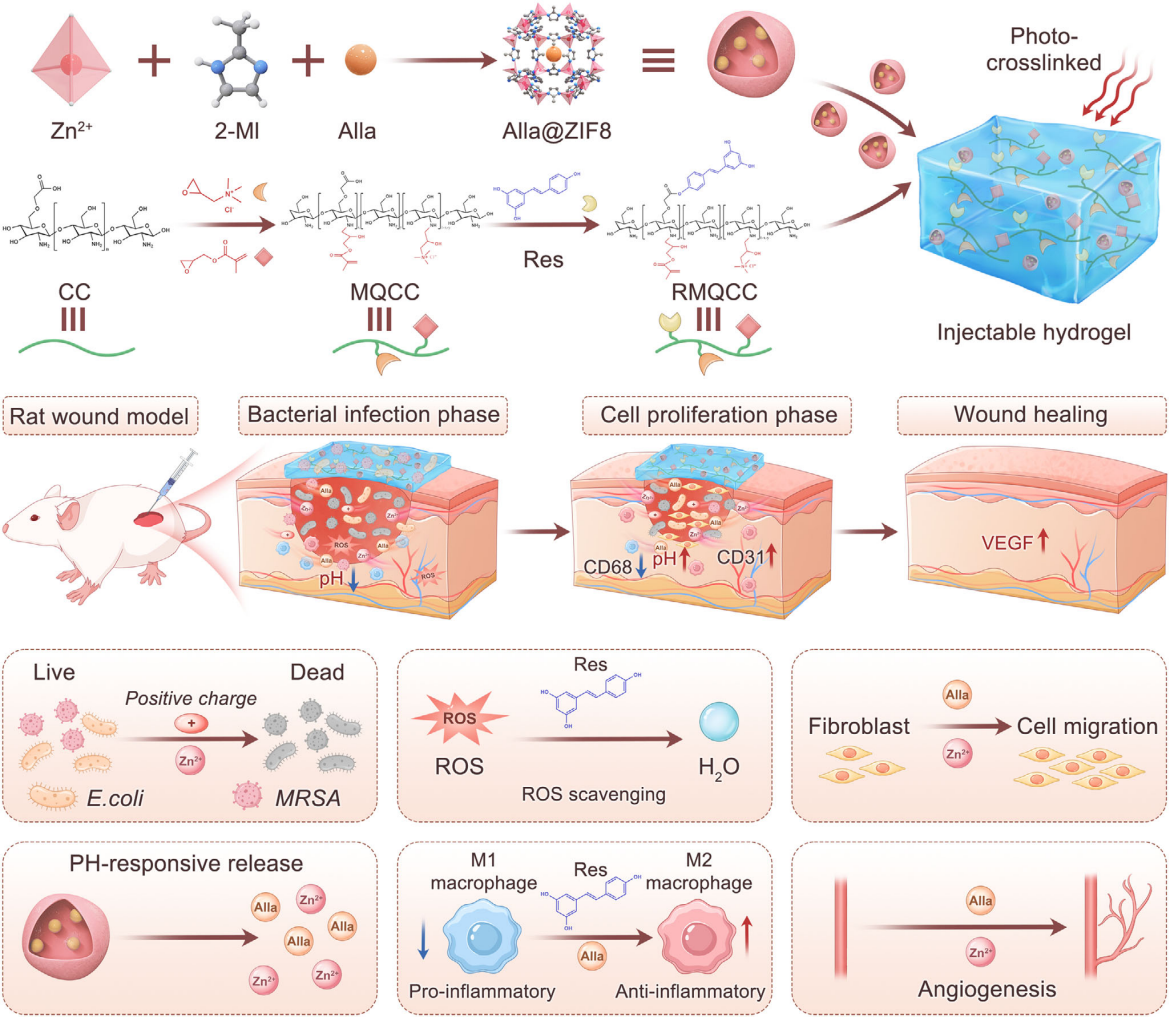
Diagram of the preparation of Alla@ZIF8‐Gel and its treatment of MRSA‐infected wounds.

In this study, a multifunctional synergistic hydrogel system (Alla@ZIF8‐Gel) was engineered by integrating Alla‐loaded ZIF8 nanoparticles (Alla@ZIF8) into a quaternary ammonium carboxymethyl chitosan hydrogel functionalized with Res (RMQCC). This design strategically amalgamates the functional advantages of each component, yielding a synergistic effect that surpasses the sum of its parts (Scheme 1). Compared to previously reported multifunctional hydrogels, the present system exhibits several distinct advantages. First, quaternization endows the hydrogel with potent antimicrobial activity, effectively suppressing wound infection while circumventing the risk of antibiotic resistance associated with conventional antimicrobial agents. Second, the presence of Res confers exceptional antioxidant properties, enabling the modulation of the oxidative stress microenvironment and thereby fostering a conducive milieu for tissue regeneration. Third, the ZIF8‐mediated Alla delivery system ensures sustained release of Alla, promoting angiogenesis and tissue regeneration. Moreover, the pH‐responsive degradation of ZIF8 nanoparticles aligns Alla release with the inflammatory status of the wound, achieving inflammation‐responsive adaptability. Finally, electrostatic interactions and hydrogen bonding impart the hydrogel with excellent injectability and self‐healing capabilities, allowing it to conform seamlessly to irregular wound geometries and establish a stable protective barrier. This multifunctional synergistic design not only addresses the limitations of single‐component materials in managing complex wound microenvironments but also achieves the integrated goals of antibacterial, antioxidant, anti‐inflammatory, and pro‐regenerative activities through component interplay. As such, it introduces a novel and highly effective therapeutic strategy for infected wound management, holding profound scientific significance and broad translational potential.

## 2 Results and Discussion

### 2.1 Preparation and Characterization of Alla@ZIF8

**FIGURE 1:**
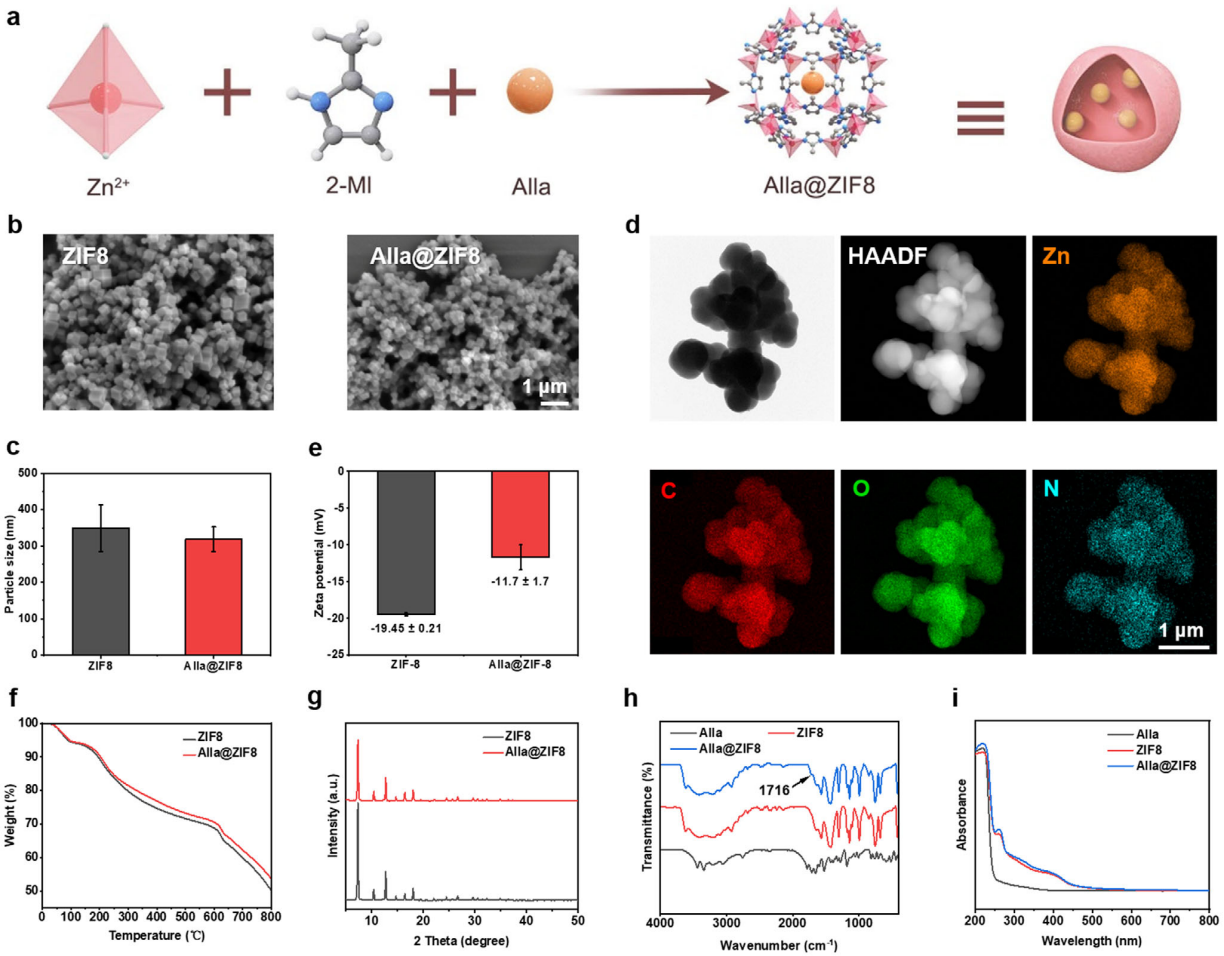
**Preparation and characterization of the Alla@ZIF8 nanoparticles**. (a) Diagram of the preparation of Alla@ZIF8 nanoparticles. (b) SEM images of ZIF8 and Alla@ZIF8 nanoparticles. (c) Particle size of ZIF8 and Alla@ZIF8 nanoparticles. (d) TEM images and element mapping images of Alla@ZIF8 nanoparticles. (e) Zeta potential of ZIF8 and Alla@ZIF8 nanoparticles. (f) TGA curves of the Zeta potential of ZIF8 and Alla@ZIF8 nanoparticles. (g) XRD patterns of ZIF8 and Alla@ZIF8 nanoparticles. (h) FTIR spectra of Alla, ZIF8, and Alla@ZIF8 nanoparticles. (i) UV–vis spectra of Alla, ZIF8 and Alla@ZIF8 nanoparticles.

The Alla@ZIF8 nanoparticles were prepared by the hydrothermal method, in which Alla was in situ loaded onto the ZIF8 framework (Figure 1a). As shown in Figure 1b, both ZIF8 and Alla@ZIF8 nanoparticles had similar polyhedral micromorphology, with average particle sizes of 349.3 and 318.5 nm, respectively, showing no statistical differences (Figure 1c). This indicated that the loading of Alla had an insignificant effect on the morphology and particle size of ZIF8. TEM further revealed that the particle size of Alla@ZIF8 was around 350 nm (Figure 1d), which was basically consistent with the result observed by SEM. Element mapping images showed that, in addition to Zn, C, and N elements, the O element derived from Alla also appeared in Alla@ZIF8, implying that Alla may be successfully loaded onto ZIF8 nanoparticles. The zeta potential results (Figure 1e) showed that, compared with ZIF8, the average surface potential of the Alla@ZIF8 increased significantly, indicating the successful loading of positively charged Alla molecules [28]. As observed TGA curves (Figure 1f), ZIF8 and Alla@ZIF8 nanoparticles exhibited similar TGA curves, and no significant difference in the mass changes between ZIF8 and Alla@ZIF8 nanoparticles, which may be attributed to the low content of Alla having a negligible influence on the ZIF8 nanoparticles. The crystal phase structure showed that the diffraction peaks of ZIF8 and Alla@ZIF8 nanoparticles were completely identical and no impurity peaks appeared; however, the intensity of the diffraction peaks of Alla@ZIF8 was significantly weaker than that of ZIF8 (Figure 1g). This indicated that the entrance of Alla into the ZIF8 framework did not alter the crystal structure of ZIF8, but decreased its crystallinity. FTIR spectra showed that compared with ZIF8, Alla@ZIF8 exhibited a weak new absorption band at 1716 cm^−1^ (Figure 1h), corresponding to the C═O stretching vibration of Alla [29], which further confirmed the successful loading of Alla. UV–vis spectra further showed that compared with ZIF8, a discriminative absorption peak at 219 nm was observed in Alla@ZIF8, also indicating the successful loading of Alla (Figure 1i).

### 2.2 Preparation and Characterization of Alla@ZIF8‐Gel

**FIGURE 2:**
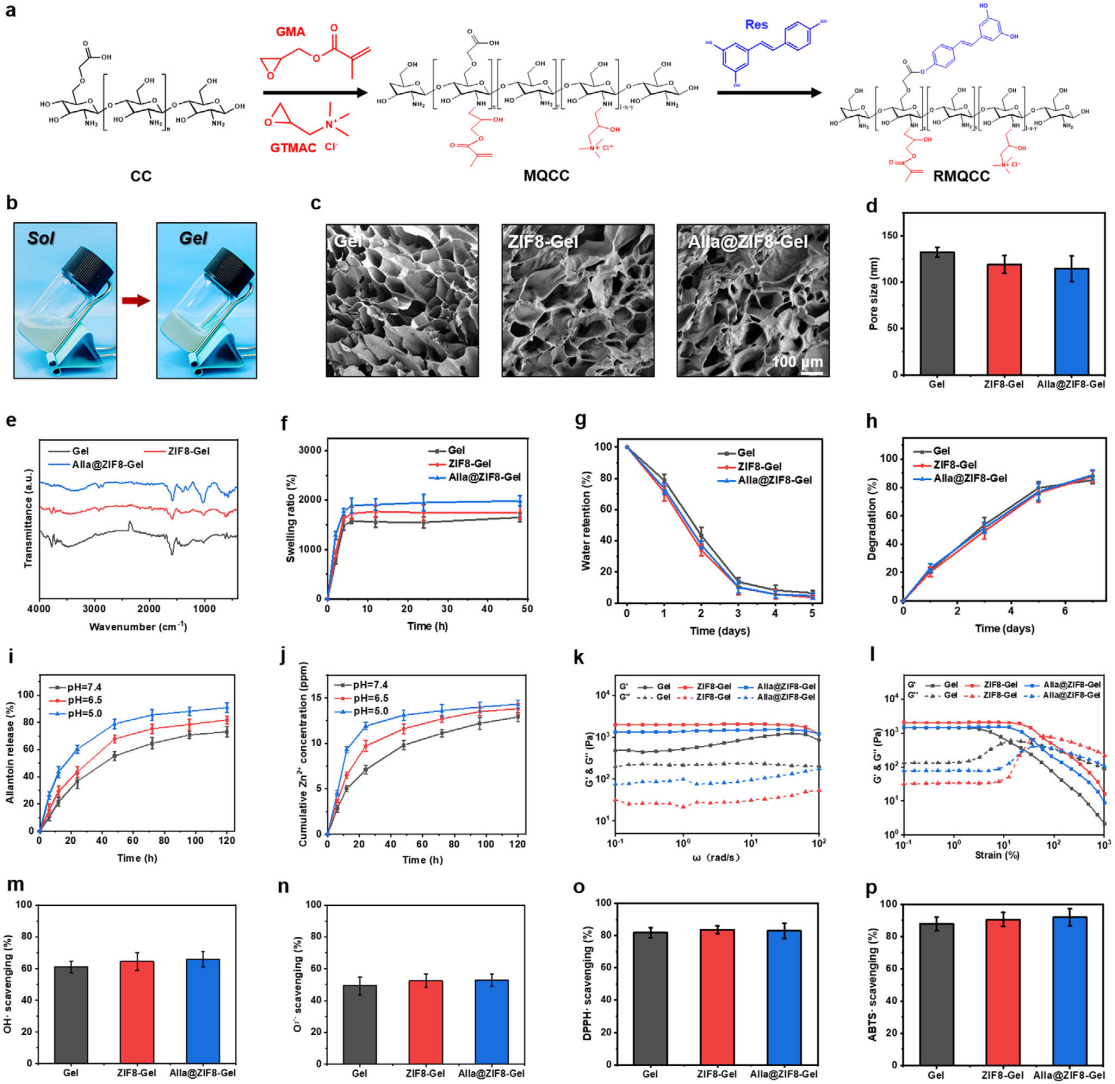
**Preparation, characterization, and properties of the nanocomposite hydrogels**. (a) The synthesis process of RMQCC. (b) Optical photographs of Alla@ZIF8‐Gel before and after light irradiation. (c) SEM images of Gel, ZIF8‐Gel and Alla@ZIF8‐Gel. (d) Pore size of Gel, ZIF8‐Gel and Alla@ZIF8‐Gel. (e) FTIR spectra of Gel, ZIF8‐Gel and Alla@ZIF8‐Gel. (f) Swelling ratio of Gel, ZIF8‐Gel and Alla@ZIF8‐Gel. (g) Water retention ratio of Gel, ZIF8‐Gel and Alla@ZIF8‐Gel. (h) Degradation ratio of Gel, ZIF8‐Gel and Alla@ZIF8‐Gel. (i) Alla release curves of Alla@ZIF8‐Gel under different pH conditions. (j) Zn^2+^ release curves of Alla@ZIF8‐Gel under different pH conditions. (k,l) Frequency sweep and strain sweep rheological tests of Gel, ZIF8‐Gel and Alla@ZIF8‐Gel. (m–p) OH·, O_2_^·−^, ABTS· and DPPH· free radical scavenging efficiency of Gel, ZIF8‐Gel and Alla@ZIF8‐Gel.

Apart from Alla@ZIF8 nanoparticles, the RMQCC matrix is also one of the fundamental components that make up the Alla@ZIF8‐Gel nanocomposite hydrogel. Herein, the RMQCC were synthesized through two‐step reactions, i.e., the epoxy ring‐opening reaction between CMCS and glycidyl methacrylate/glycidyltrimethylammonium chloride (GMA/GTMAC) and the esterification reaction between the intermediate product (MQCC) and Res (Figure 2a). ^1^H NMR (Figure S1a) showed that, compared with CMCS, some new characteristic peaks appeared in all the RMQCC. Among them, the characteristic peaks at chemical shifts δ = 5.6 and 6.1 corresponded to the C═C double bond protons in GMA, while the proton peaks at δ = 6.6∼6.9 originated from the proton signal in the Res benzene ring [30]. At δ = 3.2, the intensity of the proton peak indexed to the GTMAC quaternary ammonium group enhanced gradually with the increase of GTMAC content [31]. FTIR spectra further showed that, compared with CMCS, RMQCC exhibited three weak new absorption bands at 1654, 1604, and 1483 cm^−1^ (Figure S1b), corresponding to the stretching vibration of the C═C double bond in GMA, the stretching vibration of the C─C aromatic double bond in Res and the bending vibration of the C─H bond in quaternary ammonium group of GTMAC [30, 32, 33]. These results indicated that GMA, GTMAC, and Res successfully reacted with CMCS to obtain RMQCC. After thoroughly mixing Alla@ZIF8 nanoparticles with the RMQCC solution containing lithium phenyl (2,4,6‐trimethylbenzoyl) phosphinate (LAP) initiator and being exposed to 405 nm UV light, the Alla@ZIF8‐Gel was formed, as confirmed by the tube inversion test (Figure 2b). SEM images of the internal morphology displayed that all the hydrogels exhibited a typical interconnected porous structure (Figure 2c). Statistical results indicated that the average pore diameters of ZIF8‐Gel and Alla@ZIF8‐Gels were slightly smaller than that of Gel hydrogel (Figure 2d), which might be ascribed to the electrostatic interaction between the negatively charged nanoparticles and the positively charged amino groups in CMCS, thereby enhancing the cross‐linking density of the hydrogel network. FTIR spectra (Figure 2e) showed that, among the three hydrogel samples, apart from the characteristic absorption band of ZIF8 (1400 cm^−1^), no other significant differences were observed. This might be attributed that the content of Alla in the hydrogels is too low to exhibit the characteristic peaks.

### 2.3 Swelling, Water Retention, Degradation, Drug Release, Rheological, and Antioxidant Properties

As wound dressings, the swelling property of hydrogels is directly correlated with their ability to absorb wound exudates [34]. As shown in Figure 2f, all hydrogels exhibit rapid water uptake within the initial hour and approach swelling equilibrium within about 6 h, achieving swelling ratios ranging from 1500% to 2000%, which demonstrates their outstanding water uptake property. Good water retention capacity helps to minimize moisture evaporation from the hydrogel and maintains a moist wound environment, promoting the migration and proliferation of epithelial cells [35]. It can be seen that the water retention ratio of all the hydrogels remained above 60% after 24 h, indicating their favorable moisture‐maintaining features. Furthermore, no notable differences in water retention were observed among the three hydrogel types (Figure 2g). The degradation profile of hydrogels is also an important consideration in wound healing. A suitable degradation rate can facilitate cell migration and proliferation by creating physical space, thereby supporting tissue regeneration [36]. According to Figure 2h, all the hydrogels displayed less than 80% degradation after 5 days of immersion in PBS solution, with no significant variations among the different hydrogels.

Alla and Zn^2+^ play a beneficial role in promoting the repair and regeneration of skin tissues [37, 38]. In addition, Zn^2+^ possesses notable antibacterial activity, making it essential to investigate the release behaviors of both Alla and Zn^2+^ from the hydrogel system (Figure S2). First, the Alla loading content in ZIF‐8 nanoparticles was calculated to be 289.1 mg, with a drug loading efficiency of 82.6%. As depicted in Figure 2i, the release behavior of Alla from the Alla@ZIF8 hydrogel was consistent across neutral (pH = 7.4) and acidic (pH = 6.5 and 5.0) conditions: an initial rapid release within the first two days, followed by stabilization at the fifth day. Notably, the release rate of Alla was significantly accelerated under acidic conditions compared to neutral pH, as the cumulative Alla release over 48 h of Alla@ZIF8‐Gel at pH = 7.4, 6.5, and 5.0 was determined to be 0.93, 1.14, and 1.33 mg, respectively. A similar release trend was observed for Zn^2+^ (Figure 2j), and the 48‐h cumulative Zn^2+^ release at pH = 7.4, 6.5, and 5.0 reached 9.8, 11.6, and 13.1 ppm. Apparently, the release of both Alla and Zn^2+^ is pH‐responsive, which can be attributed that, in an acidic environment, abundant H^+^ promotes the protonation of N atoms in imidazole groups, which destroys the electrostatic attraction between Zn^2+^ and 2‐MI ligands, thereby leading to the dissociation of Zn─N coordinate bonds. As a result, the accelerated release of the loaded Alla and Zn^2+^ occurs. These findings demonstrate that the release of both Alla and Zn^2+^ from the Alla@ZIF8 hydrogel is pH‐responsive, a desirable characteristic for promoting tissue repair within the acidic pathological microenvironment typical of wounds (pH ≈ 6.5). Rheological properties are key determinants of hydrogel performance. We evaluated the rheological behavior using multiple scanning modes. As shown in Figure 2k, the storage modulus (G′) consistently exceeded the loss modulus (G″) for all the hydrogels across the entire frequency range, confirming their solid‐like elastic behavior characteristic. Specifically, the average G′ values for ZIF8‐Gel and Alla@ZIF8‐Gels reached 1208.5 and 1196.2 Pa, respectively (Figure S3a), surpassing that of the pure Gel hydrogel (849.8 Pa), indicating that nanoparticle incorporation enhanced the mechanical strength of the hydrogel network. Strain sweep measurements (Figure 2l) revealed that at low strains, G′ remained higher than G″ for all samples. With increasing strain, the intersection between G′ and G″ was observed, indicating network breakdown and a transition from gel to sol state. The critical strain values for ZIF8‐Gel and Alla@ZIF8‐Gels were significantly higher than those of the Gel hydrogel (Figure S3b). Since wounded skin often exists in a state of oxidative stress with excessive free radical generation, the antioxidant capacity of hydrogels is vital for supporting healing [39]. We therefore assessed the in vitro radical scavenging ability of the hydrogels. As shown in Figure 2m–p, all three hydrogels (Gel, ZIF8‐Gel, and Alla@ZIF8‐Gel) exhibited strong scavenging activity against reactive oxygen species (OH·, O_2_^·−^) and reactive nitrogen species (ABTS·, DPPH·), with no significant differences among them. This pronounced antioxidant effect is largely attributed to the abundance of phenolic hydroxyl groups within the resveratrol molecular structure.

### 2.4 Injectable, Self‐healing, Mechanical, and Adhesive Properties

**FIGURE 3:**
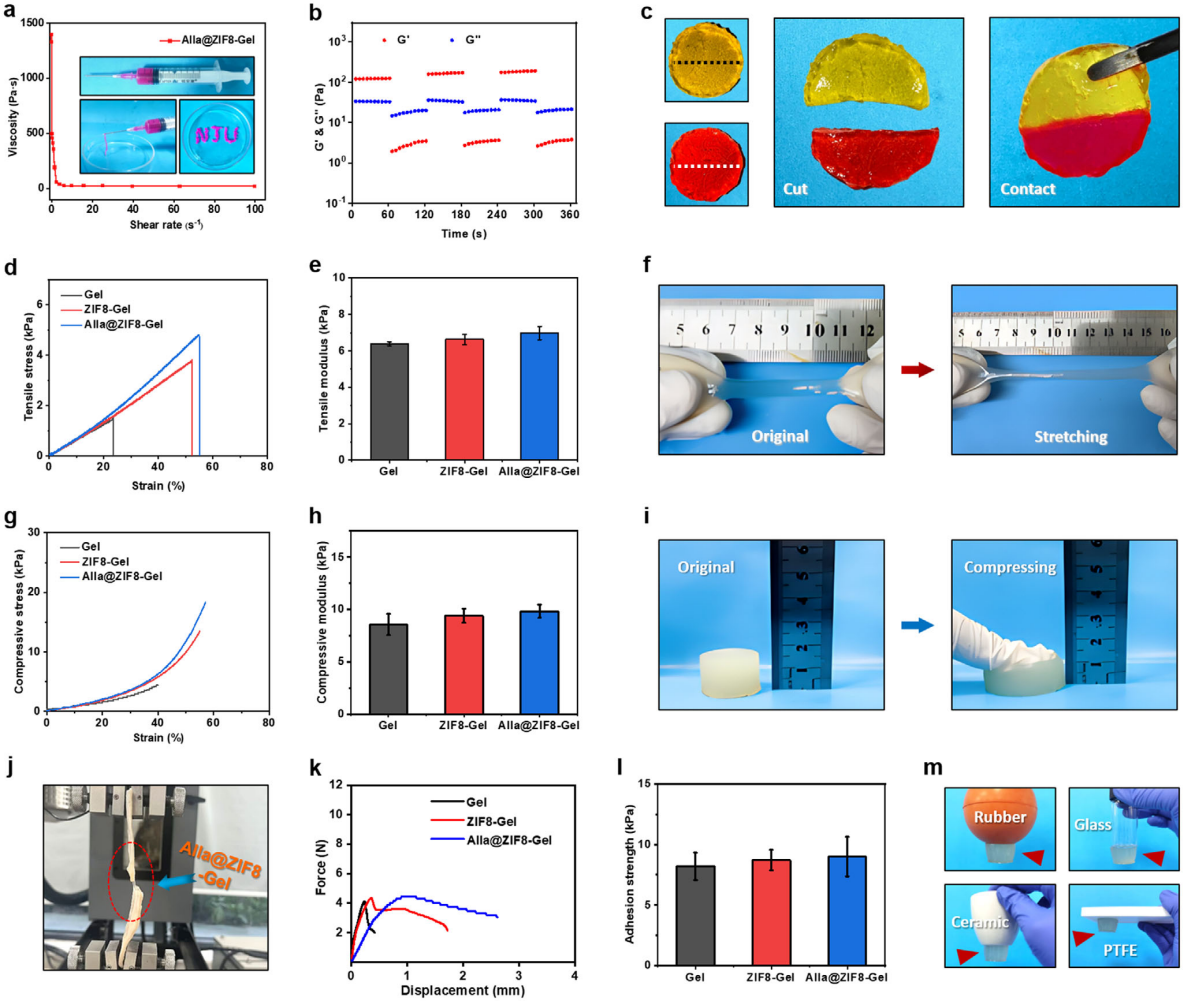
**Injectable, self‐healing, mechanical, and adhesive properties of the Alla@ZIF8‐Gel**. (a) Shear‐thinning curve Alla@ZIF8‐Gel (Inset: Display of injectability of Alla@ZIF8‐Gel with a syringe). (b) Rheological behaviors of Alla@ZIF8‐Gel with alternate strain switched from 1% to 400%. (c) Optical photographs for macroscopic self‐healing of Alla@ZIF8‐Gel. (d‐e) Tensile stress‐strain curves and tensile modulus of Gel, ZIF8‐Gel and Alla@ZIF8‐Gel. (f) Optical tensile photographs of Alla@ZIF8‐Gel. (g‐h) Compressive stress‐strain curves and compressive modulus of Gel, ZIF8‐Gel and Alla@ZIF8‐Gel. (i) Optical compressive photographs of Alla@ZIF8‐Gel. (j) Optical photographs of the adhesion test of Alla@ZIF8‐Gel. (k‐l) Force‐displacement curve and adhesion strength of Gel, ZIF8‐Gel and Alla@ZIF8‐Gel during the lap‐shear test. (m) Optical photographs of adhesion of Alla@ZIF8‐Gel to different substrate surfaces.

Injectable hydrogels are highly desirable for clinical applications due to their ability to conform to irregular wound geometries in a minimally invasive manner, thereby reducing patient discomfort. The viscosity of the Alla@ZIF8‐Gel decreased markedly as the shear rate increased from 0 to 100 s^−1^ (Figure 3a), indicating its shear‐thinning behavior. And also, the Alla@ZIF8‐Gel could be drawn into the letters “NJU” with a syringe (the inset of Figure 3a), demonstrating the favorable injectability. In practical use, hydrogels may experience structural breakdown under excessive stress, compromising functionality. Hence, the assessment of the self‐healing capability of the Alla@ZIF8‐Gel through dynamic strain alternation and static fusion assays is necessary. Under alternating strain, G′ exceeded G″ at 1% strain, whereas G″ surpassed G′ at 400% strain (Figure 3b), indicating disruption of the gel network and transition to a sol state. Subsequent reduction in strain to 1% restored G′ above G″, confirming structural recovery. Additionally, two separated hydrogel segments fused completely into an integrated structure after 1 h at 37 °C (Figure 3c), further supporting excellent self‐healing performance. The good injectable and self‐healing properties may be attributed to the high‐efficiency recombination of the reversible hydrogen bonding and electrostatic interactions between RMQCC chains and Alla@ZIF‐8 nanoparticles after external force‐induced disruption has occurred.

Appropriate mechanical properties are critical for clinical performance. Tensile testing revealed that both ZIF8‐Gel and Alla@ZIF8‐Gels exhibited significantly greater extensibility compared to the Gel hydrogel, though their tensile modulus remained comparable (Figure 3d,e, Figure S4). Visual documentation confirmed that Alla@ZIF8‐Gel samples could be stretched from approximately 5 cm to 9 cm without fracture (Figure 3f). In compression tests, all three hydrogels showed similar stress–strain responses (Figure 3g), with no statistically significant differences in compressive modulus (Figure 3h). The cylindrical Alla@ZIF8‐Gel specimens were compressed from 1.5 to 1.0 cm without visible cracking (Figure 3i), indicating robust compressive durability. Strong tissue adhesion is beneficial for sealing wounds and preventing fluid leakage and bacterial invasion [40]. An in vitro adhesion assay was performed using fresh porcine skin as a biological substrate (Figure 3j). The force–displacement curves indicated an initial rapid increase in adhesive force followed by a gradual decline for all hydrogels (Figure 3k). The mean adhesion strengths for Gel, ZIF8‐Gel, and Alla@ZIF8‐Gel were calculated as 8.2, 8.7, and 9.0 kPa, respectively (Figure 3l), with no significant differences among groups. Furthermore, the Alla@ZIF8‐Gel adhered firmly to various common materials, including rubber, glass, ceramic, PTFE, metal, and plastic (Figure 3m, Figure S5, Video S1), demonstrating broad adhesive capability. Adhesive hydrogels can achieve instantaneous wound sealing and exhibit potent haemostatic properties [41]. Given its excellent tissue adhesion, Alla@ZIF8‐Gel emerges as a promising candidate for haemostatic applications. As illustrated in Figure S6a, bloodstains on filter paper were markedly reduced for the Gel, ZIF8‐Gel, and Alla@ZIF8‐Gel groups compared to the control. Quantitative analysis of hepatic haemorrhage in a rat model revealed that the volume of blood loss was significantly lower in the Gel, ZIF8‐Gel, and Alla@ZIF8‐Gel groups relative to the untreated control. Furthermore, the recorded bleeding times demonstrated a significant reduction for all hydrogel‐treated groups, with the control group exhibiting the most prolonged haemostasis (Figure S6b,c). These findings collectively indicate that Alla@ZIF8‐Gel, possessing both robust adhesive and superior haemostatic capabilities, functions as an effective anti‐haemorrhagic hydrogel barrier, highlighting its considerable potential for practical clinical applications.

### 2.5 Biocompatibility Assessment

Exceptional biocompatibility is a fundamental prerequisite for the safe and effective therapeutic application of hydrogel dressings in infected wound management [42, 43]. By mitigating immune rejection and cytotoxicity, optimal biocompatibility establishes a conducive microenvironment for wound healing. Furthermore, it enables synergistic antimicrobial therapy, supports cellular regeneration, and fosters conditions conducive to accelerated infection control and tissue repair.

**FIGURE 4:**
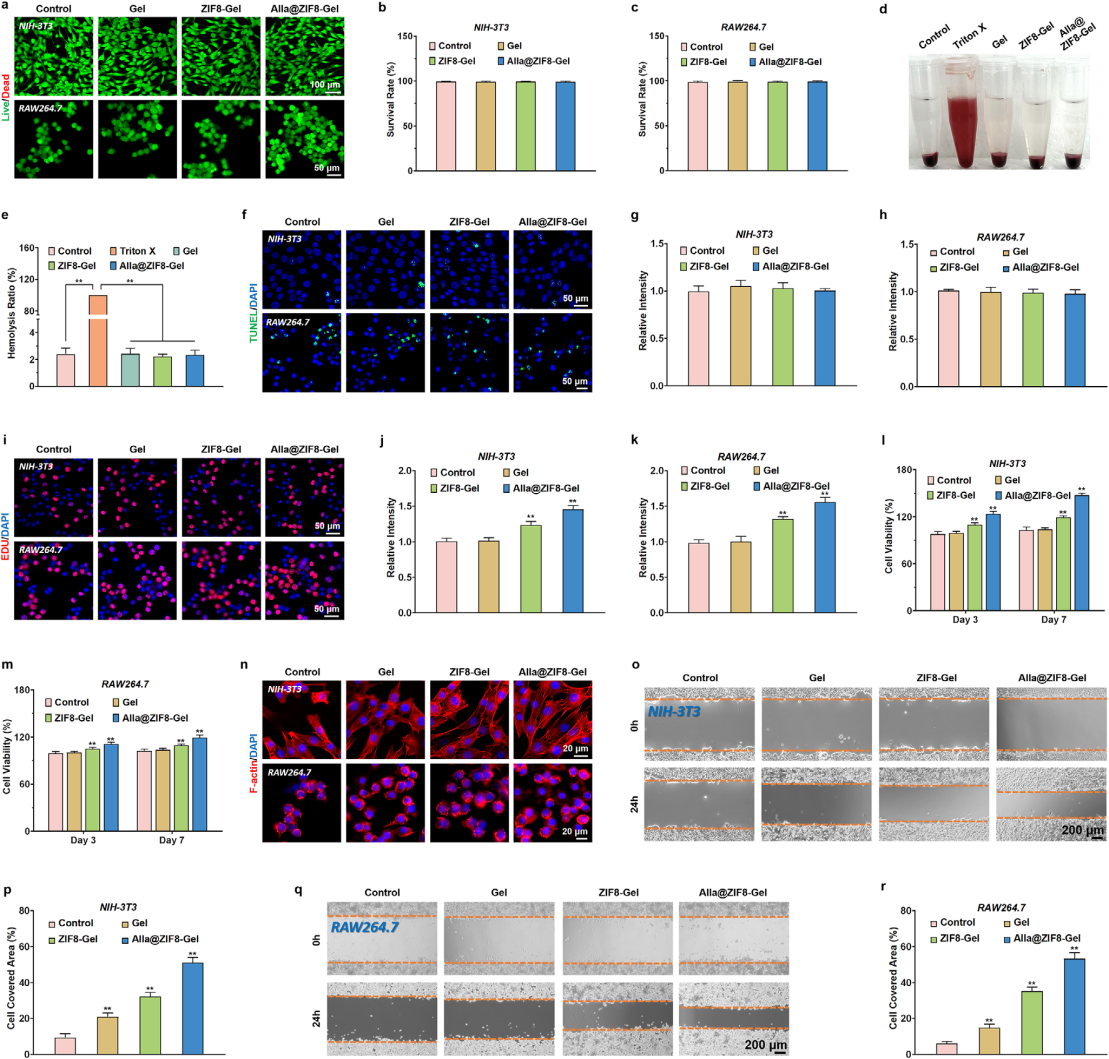
**Well Biocompatibility of the Alla@ZIF8‐Gel**. (a) Representative Live/Dead staining fluorescence images of NIH‐3T3 and RAW 264.7 cells after corresponding treatment. (b,c) Quantitative analysis of the proportion of live and dead NIH‐3T3 and RAW 264.7 cells. (d) Representative photos of blood compatibility assessment of Alla@ZIF8‐Gel. (e) Quantitative analysis of the hemolysis rate of each hydrogel. (f) Representative TUNEL staining images of NIH‐3T3 and RAW 264.7 cells after corresponding treatment. (g,h) Relative quantitative analysis of TUNEL fluorescence intensity in NIH‐3T3 and RAW 264.7 cells co‐cultured with different hydrogels. (i) Representative EDU staining images of NIH‐3T3 and RAW 264.7 cells after corresponding treatment. (j,k) Relative quantitative analysis of EDU fluorescence intensity in NIH‐3T3 and RAW 264.7 cells co‐cultured with different hydrogels. (l,m) Cell viability assessment of NIH‐3T3 and RAW 264.7 cells after 3 and 7 days of co‐culture with each hydrogel. (n) Representative fluorescent images of cytoskeleton staining of NIH‐3T3 and RAW 264.7 cells co‐cultured with each hydrogel. (o,p) NIH‐3T3 cell migration treated with different hydrogels and quantitative analysis of the coverage area of migrating cells. (q,r) RAW 264.7 cell migration treated with different hydrogels and quantitative analysis of the coverage area of migrating cells. Data are shown as the mean ± SD, *p<0.05 and **p<0.01. Statistical analysis between groups was conducted using One‐way ANOVA.

In this study, we first evaluated the cytotoxicity of Alla@ZIF8‐Gel with varying Alla@ZIF8 concentrations using Live/Dead staining. The results revealed that the viability of both NIH‐3T3 and RAW 264.7 cells remained close to 100% across all concentration groups (Figure S7). Consequently, Alla@ZIF8‐Gel containing 2.0 mg/mL Alla@ZIF8 nanoparticles was selected as the optimal formulation for subsequent biological experiments. Subsequent Live/Dead staining assays demonstrated that Gel, ZIF8‐Gel, and Alla@ZIF8‐Gel all exhibited excellent biocompatibility (Figure 4a–c). This may be attributed to the natural origin of the primary components of Alla@ZIF8‐Gel, namely, carboxymethyl chitosan, allantoin, and resveratrol, which collectively confer inherent biocompatibility. The hemocompatibility of Alla@ZIF8‐Gel was further assessed via hemolysis assays. As shown in Figure 4d, complete hemolysis was observed in the Triton X‐100 Group (Positive Control). In contrast, the red blood cell suspensions in the Gel, ZIF8‐Gel, and Alla@ZIF8‐Gel groups exhibited no significant color difference compared to the Control group. Quantitative absorbance analysis indicated that the hemolysis rates of all hydrogel groups were below 3% (Figure 4e), confirming the favorable hemocompatibility of Alla@ZIF8‐Gel. TUNEL staining further corroborated these findings, revealing no significant apoptosis in NIH‐3T3 or RAW 264.7 cells co‐cultured with Gel, ZIF8‐Gel, or Alla@ZIF8‐Gel (Figure 4f–h).

To evaluate the effect of Alla@ZIF8‐Gel on the proliferative activity of co‐cultured NIH‐3T3 and RAW 264.7 cells, EDU staining and CCK‐8 assays were performed. The results indicated that cells in the Alla@ZIF8‐Gel group exhibited robust proliferative activity after 3 and 7 days of co‐culture, further supporting the notion that Alla@ZIF8‐Gel effectively sustains cell growth, thereby creating favorable conditions for subsequent in vivo implantation (Figure 4i–m). Notably, the encapsulated Alla@ZIF8 nanoparticles did not adversely affect the overall biocompatibility of the hydrogel. Cell adhesion, a critical prerequisite for cell migration and proliferation, was investigated using rhodamine‐labeled phalloidin staining. The results demonstrated that, compared to the Control group, Gel, ZIF8‐Gel, and Alla@ZIF8‐Gel significantly enhanced the adhesion of NIH‐3T3 and RAW 264.7 cells (Figure 4n). The underlying mechanism may involve the anionic groups on the surface of carboxymethyl chitosan, which interact electrostatically with cationic moieties on cell surfaces, thereby promoting cell adhesion. Additionally, ZIF8 nanoparticles can release zinc ions, which further facilitate cell adhesion and proliferation. Alla also contributes to cell proliferation and repair, while enhancing cell‐matrix interactions. Thus, the pro‐adhesive properties of Alla@ZIF8‐Gel are likely attributable to the synergistic effects of carboxymethyl chitosan, ZIF8, and Alla.

### 2.6 Cell Migration Assay

The promotion of cell migration constitutes a core mechanism underlying the therapeutic efficacy of hydrogel dressings in treating infected wounds [44]. By facilitating the targeted recruitment of immune cells, such as macrophages and neutrophils, to the wound site, hydrogels enhance pathogen clearance and restrict infection dissemination, thereby establishing a sterile microenvironment conducive to healing. Concurrently, the moist microenvironment and bioactive components provided by hydrogels significantly promote the migration and proliferation of epithelial cells and fibroblasts, accelerating tissue regeneration, re‐epithelialization, and ultimately reducing healing time and scar formation. In this study, a scratch assay was employed to investigate the effect of Alla@ZIF8‐Gel on cell migration. As illustrated in Figure 4o,p, the scratch area in all groups progressively narrowed over time, with the most pronounced closure observed in the Alla@ZIF8‐Gel group. Quantitative analysis of cell‐covered area revealed that both the Gel and ZIF8‐Gel groups exhibited greater scratch closure than the Control group, whereas the Alla@ZIF8‐Gel group demonstrated significantly enhanced wound repair compared to both Gel and ZIF8‐Gel groups. This phenomenon may be attributed to two primary factors: first, carboxymethyl chitosan, serving as the hydrogel backbone, provides chemical cues via carboxyl (‐COOH) and quaternary ammonium (‐N^+^(CH_3_)_3_) groups that stimulate cell migration. Second, ZIF8 nanoparticles not only function as carriers for allantoin but also modulate cell migration through the sustained release of zinc ions. Additionally, Alla, a key bioactive component, directly enhances cell migration by activating intracellular signaling pathways. A similar trend was observed in scratch assays using RAW 264.7 cells (Figure 4q,r), indicating that this multi‐target, hierarchical regulatory mechanism markedly enhances cell migration efficiency. This synergistic effect further promotes the migration of both inflammatory and vascular endothelial cells, thereby fostering an accelerated wound healing process.

### 2.7 Antioxidant Damage Capacity

**FIGURE 5:**
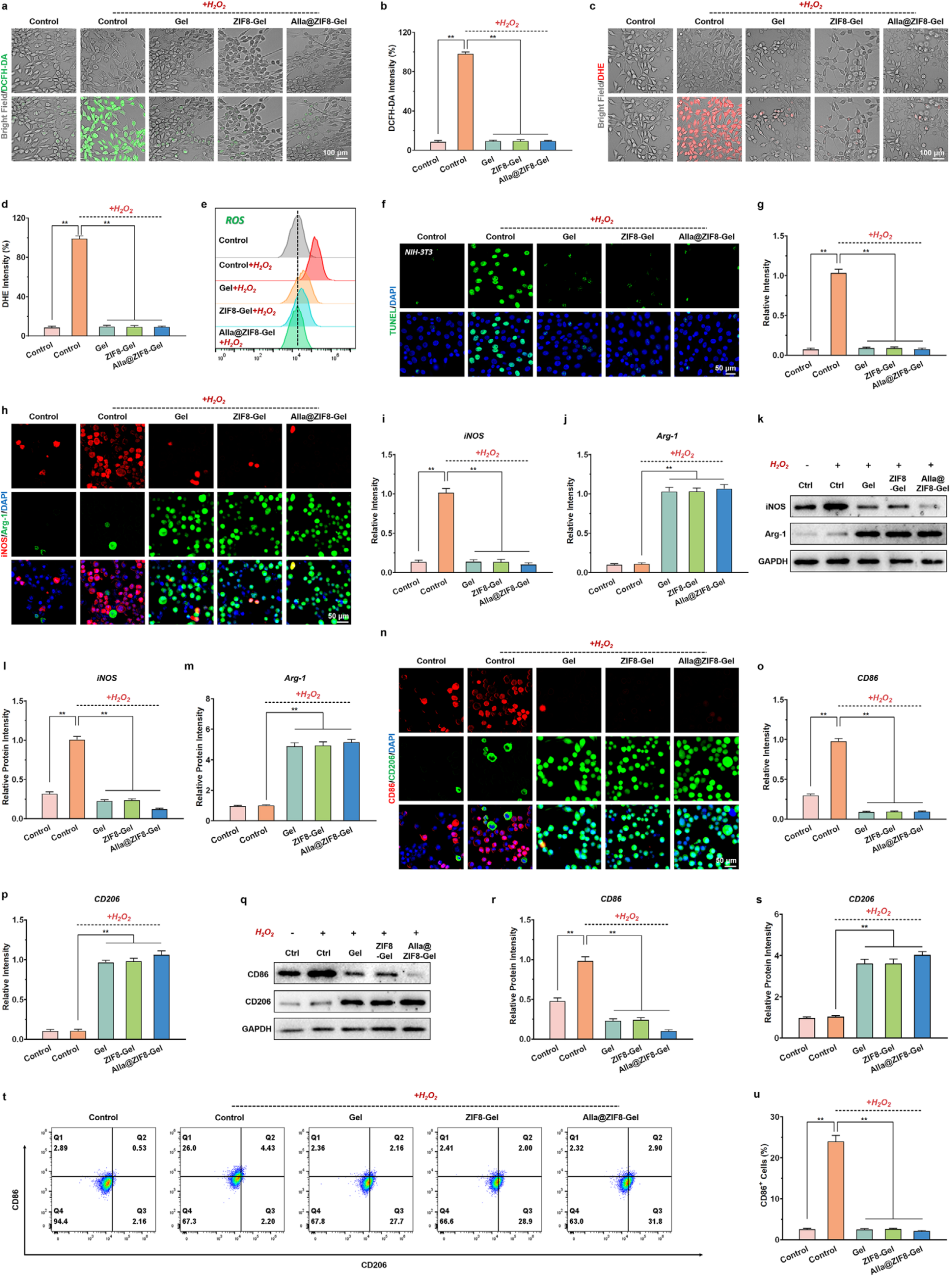
**Anti‐oxidative stress and immunomodulatory effects of the Alla@ZIF8‐Gel**. (a) Representative DCFH‐DA staining images of NIH‐3T3 cells in each group. (b) Relative DCFH‐DA fluorescence intensity analysis. (c) Representative DHE staining images of NIH‐3T3 cells in each group. (d) Relative DHE fluorescence intensity analysis. (e) Flow cytometry analysis of intracellular ROS level in different groups of NIH‐3T3 cells. (f) Representative images of TUNEL staining of NIH‐3T3 cells in each group. (g) Quantitative analysis of relative TUNEL fluorescence intensity. (h–j) Representative images and quantitative analysis of iNOS and Arg‐1 immunofluorescence staining of RAW 264.7 cells in each group under oxidative stress microenvironment. (k–m) Protein expression of iNOS and Arg‐1 in RAW 264.7 cells co‐cultured with each hydrogel. (n–p) Representative images and quantitative analysis of CD86 and CD206 immunofluorescence staining of RAW 264.7 cells in each group under oxidative stress microenvironment. (q–s) Protein expression of CD86 and CD206 in RAW 264.7 cells co‐cultured with each hydrogel. (t) Flow cytometry results of CD86 and CD206 in each group of RAW 264.7 cells. (u) Quantitative analysis of CD86 positive RAW 264.7 cell percentage detected by flow cytometry in each group. Data are shown as the mean ± SD, *p<0.05 and **p<0.01. Statistical analysis between groups was conducted using One‐way ANOVA.

Previous studies have demonstrated that antioxidant hydrogels can protect key reparative cells, such as fibroblasts, from oxidative damage by scavenging excess ROS accumulated in wound tissues [45]. This action preserves their normal proliferative and migratory functions, thereby preventing healing delays caused by lipid peroxidation and DNA damage. To evaluate the performance of Alla@ZIF8‐Gel in mitigating oxidative stress injury, NIH‐3T3 cells were co‐cultured with the respective hydrogels under a simulated high oxidative stress microenvironment, followed by DCFH‐DA, DHE, and TUNEL staining assays. The results of DCFH‐DA and DHE staining revealed intense green and red fluorescence signals within NIH‐3T3 cells in the Control + H_2_O_2_ group, indicative of severe oxidative damage (Figure 5a–d). In contrast, ROS levels were markedly reduced in NIH‐3T3 cells co‐cultured with Gel, ZIF8‐Gel, and Alla@ZIF8‐Gel. This phenomenon confirms that the Res in Alla@ZIF8‐Gel confers potent antioxidant capacity, consistent with prior in vitro free radical scavenging experiments. Flow cytometry analysis of intracellular ROS levels yielded similar results, further corroborating that Alla@ZIF8‐Gel effectively alleviates oxidative damage in epithelial cells through ROS scavenging (Figure 5e, Figure S8). Furthermore, TUNEL staining showed a substantial number of apoptotic NIH‐3T3 and RAW 264.7 cells in the Control + H_2_O_2_ group, whereas fewer apoptotic cells were observed in the Gel + H_2_O_2_, ZIF8‐Gel + H_2_O_2_, and Alla@ZIF8‐Gel + H_2_O_2_ groups, suggesting that Alla@ZIF8‐Gel suppresses epithelial cell apoptosis via its antioxidant effects (Figure 5f–g, Figure S9).

### 2.8 Macrophage Polarization Regulation

Modulation of the inflammatory response, achieved by significantly reducing the expression of pro‐inflammatory cytokines such as TNF‐α and IL‐6 while elevating levels of anti‐inflammatory cytokines like IL‐10, can effectively mitigate excessive inflammation at the wound site [46]. This creates a favorable immune microenvironment for tissue regeneration, thereby accelerating infection control and tissue repair [47]. Furthermore, regulating macrophage polarization, specifically, promoting the transition from pro‐inflammatory M1 to anti‐inflammatory M2 phenotypes, can abbreviate the inflammatory phase and synergistically enhance angiogenesis, collagen deposition, and re‐epithelialization, ultimately restoring the structural and functional integrity of the wound tissue [48]. To investigate the impact of Alla@ZIF8‐Gel on macrophage polarization under high oxidative stress conditions, a comprehensive approach was employed, including immunofluorescence staining, RT‐qPCR, western blotting (WB), flow cytometry, and enzyme‐linked immunosorbent assay (ELISA) to assess the expression of macrophage subtype markers. Classical markers for M1 macrophages include iNOS and CD86, whereas Arg‐1 and CD206 are characteristic of M2 macrophages. Immunofluorescence staining revealed that, compared with the Control group, the Gel, ZIF8‐Gel, and Alla@ZIF8‐Gel groups markedly decreased iNOS expression while enhancing Arg‐1 fluorescence signals in RAW 264.7 cells, indicating effective promotion of macrophage polarization toward the M2 phenotype (Figure 5h–j). As shown in Figure 5k–m, WB results were highly consistent with immunofluorescence findings, further confirming that Alla@ZIF8‐Gel alleviates inflammation at the injury site by suppressing M1 macrophage polarization. Additionally, both immunofluorescence and WB analyses demonstrated that Alla@ZIF8‐Gel significantly reduced CD86 expression while increasing CD206 fluorescence signals in RAW 264.7 cells (Figure 5n–s). RT‐qPCR and flow cytometry data corroborated these observations (Figure 5t–u, Figure S10 and S11). Moreover, ELISA results revealed that RAW 264.7 cells co‐cultured with Gel, ZIF8‐Gel, and Alla@ZIF8‐Gel exhibited significantly elevated secretion of M2‐associated anti‐inflammatory cytokines and markedly reduced pro‐inflammatory cytokine release (Figure S12). Notably, Alla@ZIF8‐Gel induced a greater proportion of M2‐polarized macrophages and fewer M1‐polarized cells compared to Gel and ZIF8‐Gel. The underlying mechanism may involve the inherent capacity of Alla to enhance macrophage phagocytic activity and drive their transition toward an anti‐inflammatory phenotype. Additionally, Alla can suppress the activation of canonical pro‐inflammatory pathways, such as NF‐κB and MAPK, thereby significantly downregulating pro‐inflammatory cytokine expression in macrophages. Collectively, these data demonstrate that Alla@ZIF8‐Gel, through ROS scavenging combined with the immunomodulatory functions of Alla, effectively promotes M2 macrophage polarization under high oxidative stress conditions, thereby alleviating inflammation in infected wound tissues.

### 2.9 Antibacterial Activity In Vitro

**FIGURE 6:**
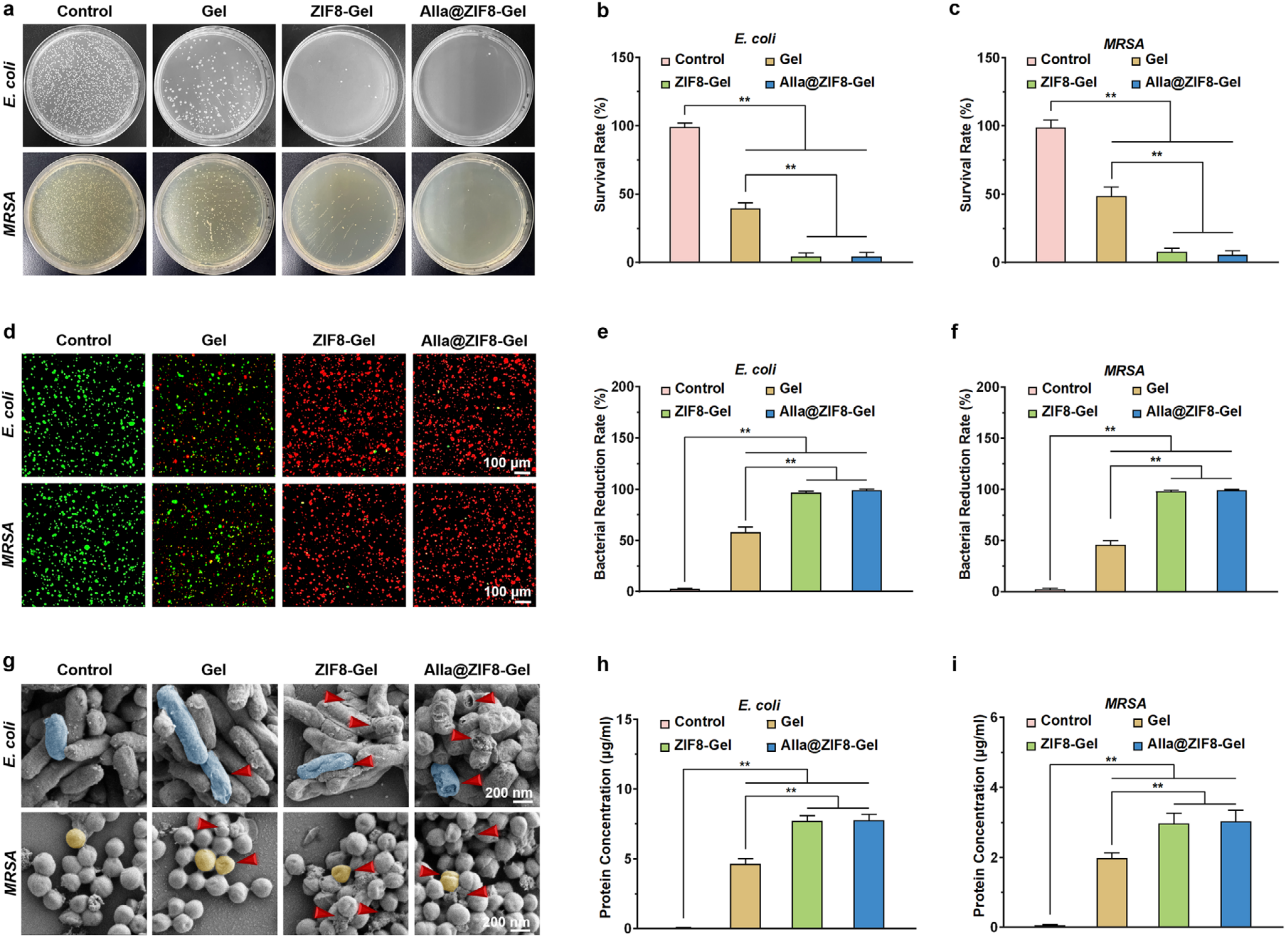
**Well antibacterial performance of the Alla@ZIF8‐Gel in vitro**. (a) Representative CFU photos of *E. coli* and *MRSA* on agar plates after corresponding treatment. (b) Quantitative analysis of the bacterial survival rate of *E. coli* in each group. (c) Quantitative analysis of the bacterial survival rate of *MRSA* in each group. (d) Representative images of SYTO‐9/PI staining of *E. coli* and *MRSA* after co‐culture with each hydrogel. (e) Quantitative analysis of bacterial reduction rate of *E. coli* in each group. (f) Quantitative analysis of bacterial reduction rate of *MRSA* in each group. (g) Representative SEM images of *E. coli* and *MRSA* morphology after corresponding treatment. The red arrow indicated the cracked and dead bacteria. (h,i) Quantitative analysis of leakage proteins from *E. coli* and *MRSA* after different treatments. Data are shown as the mean ± SD, *p<0.05 and **p<0.01. Statistical analysis between groups was conducted using One‐way ANOVA.

A central challenge in the management of infected wounds is the effective eradication of pathogenic bacteria [49]. Hydrogel dressings with robust antibacterial properties can markedly suppress microbial colonization, significantly reduce bacterial load at the wound site, and prevent infection‐induced tissue necrosis and inflammatory cascades, thereby establishing a sterile microenvironment conducive to subsequent healing [50]. Concurrently, potent antibacterial activity can abbreviate the inflammatory phase, mitigate damage to reparative cells by bacterial toxins, and synergize with the pro‐angiogenic and collagen‐depositing functions of the hydrogel to accelerate re‐epithelialization and functional tissue restoration [51]. Experimental results revealed that, compared with the Control group, the Gel group exhibited a substantial reduction in the survival rates of *MRSA* and *E. coli* (Figure 6a–c), as evidenced by fewer CFU on respective agar plates. This effect is attributable to the quaternized carboxymethyl chitosan within the hydrogel matrix, whose high‐density cationic groups (‐N^+^(CH_3_)_3_) engage in strong electrostatic interactions with anionic components of the bacterial outer membrane, such as lipopolysaccharides (LPS) and teichoic acids, resulting in membrane depolarization and increased permeability. Notably, Alla@ZIF8‐Gel demonstrated bacteriostatic rates exceeding 95% against both *MRSA* and *E. coli*, outperforming both the Control and Gel groups. This superior efficacy arises from the ZIF8 nanoparticles within Alla@ZIF8‐Gel, which act as zinc ion carriers, gradually releasing Zn^2^^+^ in the bacterial microenvironment. The released Zn^2^^+^ inhibits bacterial energy metabolism by binding to sulfhydryl groups in respiratory chain enzymes, while also coordinating with carboxyl/phosphate groups in membrane proteins, exacerbating the loss of membrane fluidity and thereby enhancing antibacterial performance. SYTO‐9/PI staining assays yielded results consistent with those from agar plate experiments. Compared with the Control, the Gel group showed greater bacterial killing of both *MRSA* and *E. Coli* (Figure 6d–f). Meanwhile, intense red fluorescence was observed within bacteria in the ZIF8‐Gel and Alla@ZIF8‐Gel groups, indicating that Alla@ZIF8‐Gel achieves highly synergistic inhibition of both Gram‐positive (*MRSA*) and Gram‐negative (*E. coli*) bacteria through the integration of the physical bactericidal action of quaternized chitosan and the ion‐disruptive effects of ZIF8. Concentration‐dependent experiments further demonstrated that the antibacterial efficacy of Alla@ZIF8‐Gel intensified with increasing concentrations of Alla@ZIF8 nanoparticles. At a concentration of 2.0 mg/mL, bacterial survival rates dropped below 5%, corresponding to a bacterial reduction rate approaching 100% (Figures S13 and S14). SEM imaging revealed that *E. coli* in the control group retained its characteristic rod‐shaped morphology, while MRSA displayed its typical grape‐like cluster arrangement (Figure 6g). In contrast, both MRSA and E. coli in the ZIF8‐Gel and Alla@ZIF8‐Gel groups underwent significant morphological deformation, featuring visible cell wall fractures, collapse, and leakage of intracellular contents. These structural alterations strongly suggest that ZIF8‐Gel and Alla@ZIF8‐Gel inflict substantial damage on these bacterial strains. Furthermore, measurements of intracellular protein and potassium ion content revealed substantial leakage of both components in the Alla@ZIF8‐Gel group (Figure 6h,i, Figure S15), corroborating that rapid bactericidal activity is achieved through disruption of bacterial membrane integrity.

### 2.10 Wound Healing in MRSA‐Infected Skin Defect Models

**FIGURE 7:**
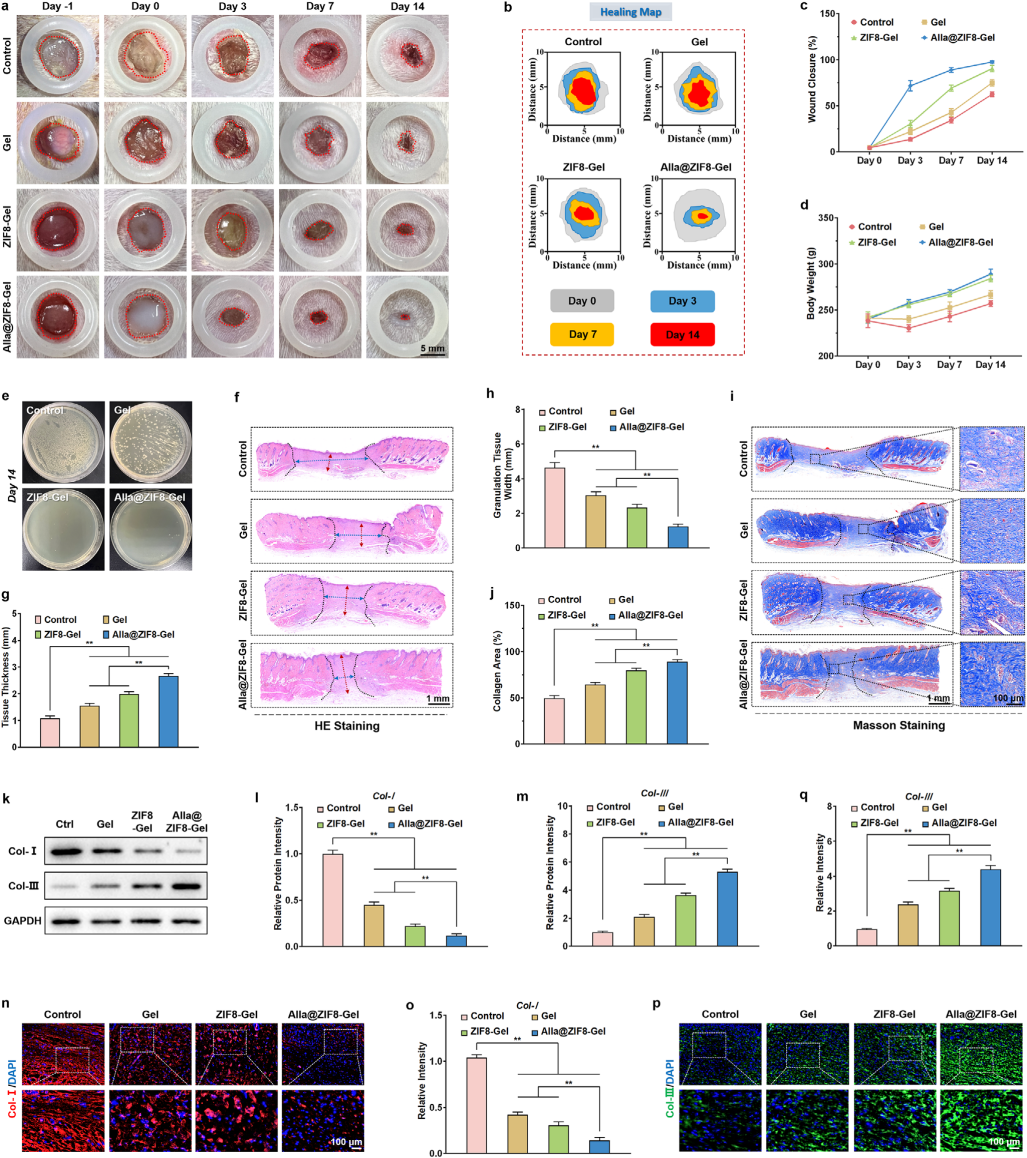
**The promoting effect of the Alla@ZIF8‐Gel on wound healing in *MRSA*‐infected rats**. (a) Representative photographs of *MRSA*‐infected wounds at different times during the treatment with each hydrogel. (b) Schematic diagram of wound closure traces during the whole treatment period. (c) Quantitative analysis of wound closure rate in rats treated with different hydrogels from day 0 to day 14. (d) The weight change of rats in each group throughout the entire treatment process. (e) *MRSA* infection in rat wounds after 14 days of treatment with different hydrogels evaluated by agar plate coating. (f) Representative HE staining images of healed wound tissue in rats from different groups on day 14. (g,h) Quantitative analysis of tissue thickness and tissue width on the 14th day of infected wounds in each group. (i) Representative Masson staining images of healed wound tissue in rats on day 14. (j) Quantitative analysis of collagen area measured from Masson staining images. (k) Representative Western blotting images of Col‐I and Col‐III protein in wound healing tissue of rats in each group. (m,n) Quantitative analysis of relative protein expression of Col‐I and Col‐III. (n,o) Representative images and quantitative analysis of Col‐I immunofluorescence on the 14th day of *MRSA*‐infected wounds in each group. (p,q) Representative images and quantitative analysis of Col‐III immunofluorescence on the 14th day of *MRSA*‐infected wounds in each group. Data are shown as the mean ± SD, *p<0.05 and **p<0.01. Statistical analysis between groups was conducted using One‐way ANOVA.

The healing of infected wounds is a complex physiological process involving multiple stages, including inflammatory regulation, tissue regeneration, and remodeling [52]. Chronic infected wounds, however, often exhibit delayed healing due to excessive inflammatory responses and bacterial biofilm formation [53]. In this study, we established an in vivo *MRSA*‐infected wound model to evaluate the antibacterial efficacy of Alla@ZIF8‐Gel and its capacity to promote wound healing. Briefly, full‐thickness circular wounds (1 cm in diameter) were created on the dorsum of rats, followed by local inoculation with an *MRSA* suspension [54]. After 24 h, agar plate culture confirmed successful MRSA colonization in all wounds prior to treatment (Figure S16). Photographic documentation of wound progression revealed a gradual reduction in wound area across all groups (Control, Gel, ZIF8‐Gel, and Alla@ZIF8‐Gel) over time (Figure 7a,b). Quantitative analysis of wound closure rates demonstrated that both Gel and ZIF8‐Gel facilitated tissue regeneration and wound closure, indicating that the favorable microenvironment provided by carboxymethyl chitosan and Res, combined with the incorporated ZIF8 nanoparticles, contributed to accelerated healing to some extent (Figure 7c). Notably, rats treated with Alla@ZIF8‐Gel exhibited the most rapid wound closure, a superior outcome attributable to the synergistic effects of its multifunctional components. First, quaternized carboxymethyl chitosan exerted potent bactericidal activity by disrupting bacterial membrane integrity through its cationic properties, thereby effectively eliminating pathogens and minimizing interference with the healing process. Second, ZIF8 nanoparticles responsively released zinc ions within the wound microenvironment, not only enhancing antibacterial performance but also promoting extracellular matrix remodeling via activation of zinc‐dependent metalloproteinases [55]. The strong antioxidant activity of Res efficiently scavenged accumulated ROS, mitigating oxidative damage to fibroblasts and endothelial cells, and further fostering collagen deposition and angiogenesis. Alla, acting as a key immunomodulator in concert with Res, induced macrophage polarization toward the M2 phenotype, thereby alleviating excessive inflammation and establishing a regenerative microenvironment [56]. Moreover, unlike rats in other groups that experienced initial weight loss during the early phase of infection, those in the ZIF8‐Gel and Alla@ZIF8‐Gel groups maintained stable weight gain throughout the experimental period (Figure 7d), further corroborating the potent antibacterial and anti‐inflammatory properties of these formulations. Consistent with the aforementioned in vivo findings, agar plate assays of treated wounds revealed a significant reduction in bacterial load in the ZIF8‐Gel and Alla@ZIF8‐Gel groups, confirming the efficacy of these treatments in resolving infection (Figure 7e).

Wound healing is a highly intricate and tightly coordinated process, encompassing a series of pathological events including wound contraction, granulation tissue formation, collagen deposition, scar formation, and re‐epithelialization [57, 58]. To investigate the histological effects of Alla@ZIF8‐Gel on rat wounds, we performed HE, Masson trichrome, and immunofluorescence staining for collagen type I (Col‐I) and collagen type III (Col‐III) on wound tissues harvested 14 days post‐treatment. HE staining revealed that Alla@ZIF8‐Gel‐treated wounds exhibited greater tissue thickness compared with the Control, Gel, and ZIF8‐Gel groups (Figure 7f). Quantitative analysis of granulation tissue width showed values of 4.6405, 3.0465, 2.3455, and 1.2476 mm for the Control, Gel, ZIF8‐Gel, and Alla@ZIF8‐Gel groups, respectively. Notably, Alla@ZIF8‐Gel treatment resulted in significantly narrower granulation tissue relative to the other groups (Figure 7g,h), likely attributable to the favourable microenvironment and potent antibacterial activity provided by the hydrogel. Furthermore, Alla@ZIF8‐Gel promoted wound healing by alleviating oxidative stress and inducing macrophage polarization toward the M2 phenotype. The combined effect of these factors explains the accelerated wound closure and superior healing outcomes observed in the Alla@ZIF8‐Gel group. Collagen deposition and its orderly arrangement are critical in infected wound healing, providing structural support for nascent tissue and facilitating fibroblast proliferation, thereby enhancing granulation tissue formation and wound closure. Moreover, collagen deposition strengthens the wound's resistance to infection, modulates inflammatory responses, and supports tissue regeneration and functional recovery [59, 60]. Masson staining of wound sections revealed that Alla@ZIF8‐Gel‐treated wounds displayed the most abundant collagen deposition and pronounced extracellular matrix (ECM) remodeling compared with the Control, Gel, and ZIF8‐Gel groups (Figure 7i,j). This outcome is likely due to the ability of Alla@ZIF8‐Gel to promote fibroblast proliferation and migration during the early stages of healing, thereby accelerating collagen synthesis and tissue repair. Previous studies have established that Col‐I is a major component of the ECM during scar formation and plays a pivotal role in skin repair. In contrast, Col‐III is also crucial for wound healing and tissue regeneration, but its expression level is closely associated with scarless wound healing. In this study, we assessed the expression levels of Col‐I and Col‐III in healed wounds via WB and immunofluorescence staining. The results demonstrated that Alla@ZIF8‐Gel significantly downregulated Col‐I expression while upregulating Col‐III (Figure 7k–q), indicating substantial suppression of scar formation in Alla@ZIF8‐Gel‐treated wounds. These findings further corroborate the potential of Alla@ZIF8‐Gel to minimize scarring during the healing of infected wounds.

### 2.11 The Mechanism of Alla@ZIF8‐Gel Promoting Infected Wound Repair

**FIGURE 8:**
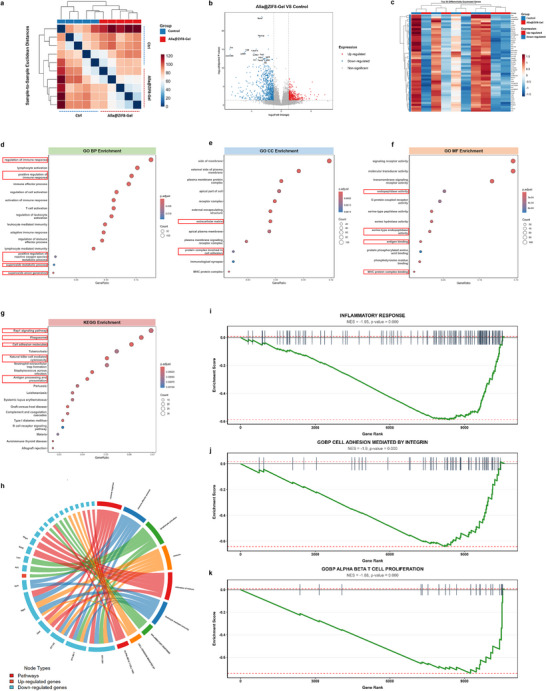
**Transcriptome sequencing analysis of wound tissues from *MRSA*‐Infected rats with and without Alla@ZIF8‐Gel treatment**. (a) Heatmap of sample‐to‐sample distances. (b) Volcano plot of the transcriptomic analysis of the differential genes between the Control and Alla@ZIF8‐Gel group. (c) Heatmap of the differential genes between the Control and Alla@ZIF8‐Gel group. (d–f) GO enrichment analysis of DEGs after Alla@ZIF8‐Gel treatment, including subgroups biological process (BP), cellular component (CC) and molecular function (MF). (g) Bubble map of the KEGG enrichment analysis. (h) Pathway‐gene network chord diagram of DEGs after Alla@ZIF8‐Gel treatment. (i–k) GSEA of the global differences in Inflammatory Response, Integrin‐mediated Cell Adhesion and T Cell Proliferation between the Control and Alla@ZIF8‐Gel group.

To elucidate the specific mechanisms by which Alla@ZIF8‐Gel promoted the repair of infected wound tissues, this study performed transcriptome sequencing on wound tissues from *MRSA*‐infected rats treated with or without Alla@ZIF8‐Gel. Principal component analysis (PCA) revealed strong intra‐group clustering and significant inter‐group differences (Figure S17). Heatmap of sample‐to‐sample distances demonstrated high reproducibility and consistency across biological replicates (Figure 8a). Differentially expressed genes (DEGs) were selected based on established thresholds. A volcano plot displayed the transcriptional profile following Alla@ZIF8‐Gel treatment, identifying 1157 significantly DEGs, including 365 upregulated and 792 downregulated genes (Figure 8b). Furthermore, a hierarchical clustering heatmap of the Top 50 DEGs clearly illustrated distinct expression patterns and sample clustering between the Control and Alla@ZIF8‐Gel group (Figure 8c). The heatmap revealed a bimodal regulatory pattern, with downregulated gene clusters predominantly on the left (blue region) and upregulated clusters on the right (red region), reflecting the multifaceted action of Alla@ZIF8‐Gel. Gene Ontology (GO) biological process (BP) enrichment analysis uncovered core molecular mechanisms through which Alla@ZIF8‐Gel facilitates wound repair (Figure 8d). Significant enrichment of terms such as “Regulation of immune response,” “Lymphocyte activation,” and “Immune effector process” highlights the dual antibacterial and anti‐inflammatory roles of Alla@ZIF8‐Gel, namely, the activation of immune cells via zinc ion release to exert antibacterial effects, while concurrently fine‐tuning immune responses to prevent excessive inflammation. Enrichment of “Positive regulation of reactive oxygen species metabolic process,” “Superoxide metabolic process,” and “Superoxide anion generation” directly confirms the unique antioxidant capacity of Alla@ZIF8‐Gel, which protects cells from oxidative stress by systematically activating reactive oxygen species scavenging systems. Cellular component (CC) enrichment analysis elucidated the precise subcellular targeting and localization of ZIF8‐based hydrogel activity (Figure 8e). Significant enrichment of “Extracellular matrix” and “Protein complex involved in cell adhesion” suggests that Alla@ZIF8‐Gel supports tissue reconstruction and structural integrity by promoting collagen deposition, modulating extracellular matrix composition, and enhancing cell adhesion. Molecular function (MF) enrichment analysis clarified the regulatory mechanisms of Alla@ZIF8‐Gel at the protein functional level (Figure 8f). Enrichment of “Endopeptidase activity” and “Serine‐type peptidase activity” reflects its role in promoting collagen deposition through the regulation of protease activity, thereby controlling extracellular matrix remodeling and collagen fibril formation. Additionally, enrichment of “Antigen binding” and “MHC protein complex binding” indicates that Alla@ZIF8‐Gel may exert antibacterial effects via antigen recognition and modulation of immune responses.

KEGG (Kyoto Encyclopedia of Genes and Genomes) enrichment analysis revealed the key cellular signaling networks activated by Alla@ZIF8‐Gel (Figure 8g). Significant enrichment of the “Rap1 signaling pathway” supports its role in promoting cell migration and angiogenesis, as Rap1, a critical small GTPase, regulates cell adhesion, migration, and endothelial cell function [61]. Enrichment of the “Phagosome” pathway underscores the antibacterial mechanism of Alla@ZIF8‐Gel, potentially enhancing phagocyte function to clear pathogens. The enrichment of cell adhesion molecule‐related pathways further corroborates its pro‐migratory effects, facilitating tissue reconstruction by modulating cell–cell and cell–matrix interactions. Involvement of “Natural killer cell mediated cytotoxicity” and related immune pathways supports anti‐inflammatory regulation, maintaining tissue homeostasis through immune cell modulation. A pathway‐gene interaction network chord diagram illustrates the complex molecular interplay underlying Alla@ZIF8‐Gel's action, highlighting its systemic, multi‐mechanistic integration (Figure 8h). The dense network of inter‐pathway connections reflects extensive gene sharing and functional synergy, providing a molecular basis for the material's simultaneous antibacterial, anti‐inflammatory, and antioxidant activities. Gene Set Enrichment Analysis (GSEA) provided detailed validation and dynamic characterization of Alla@ZIF8‐Gel's pro‐repair mechanisms, revealing precise regulatory patterns across three key pathways (Figure 8i–k). The “Inflammatory response” curve (NES = −1.96) robustly confirms anti‐inflammatory efficacy, with a sustained downward trend indicating systemic downregulation of inflammation‐related genes. This suppression effectively limits the intensity and scope of inflammation, creating an optimal microenvironment for the transition from the inflammatory to the reparative phase. The “Cell adhesion mediated by integrin” curve (NES = −1.94) supports pro‐migratory activity, as its distinctive pattern reflects fine‐tuned regulation of integrin‐mediated adhesion, critical for endothelial cell migration during angiogenesis. The “T cell proliferation” curve (NES = −1.86) indicates precise modulation of immune cell proliferation, potentially linked to antibacterial function through maintenance of T cell activation homeostasis. All three curves exhibited strong negative enrichment with high significance, demonstrating Alla@ZIF8‐Gel's capacity to exert antioxidant protection, promote collagen deposition, and orchestrate tissue repair via a tightly controlled molecular network. Normalized Enrichment Score analysis further revealed the regulatory strength and precision of Alla@ZIF8‐Gel across multiple pathways, underscoring its unique therapeutic profile (Figure S18). The strong negative enrichment of “Inflammatory response” confirms potent anti‐inflammatory activity, achieved through systemic suppression of excessive inflammation to foster a pro‐regenerative microenvironment. Negative enrichment in “Blood vessel maturation” and “Angiogenesis” suggests that Alla@ZIF8‐Gel fine‐tunes the angiogenic process, possibly by downregulating inhibitors to optimize neovascularization. Similarly, negative enrichment in “ECM receptor interaction” supports enhanced collagen deposition, reflecting precise modulation of ECM‐cell crosstalk to optimize matrix remodeling. Quantitative analysis of material‐attribute gene activation clearly delineates the molecular foundation of Alla@ZIF8‐Gel's therapeutic functions (Figure S19). “ECM remodeling” exhibited the highest number of significantly activated genes (13), strongly supporting its role in collagen deposition and matrix reorganization, core mechanisms underlying tissue regeneration and structural restoration. “Antibacterial” function activated six key genes, directly reflecting the combined action of quaternary ammonium chitosan hydrogel and zinc ions in sustaining antimicrobial effects through regulation of antimicrobial peptides, immune factors, and pathogen recognition genes. “Anti‐inflammatory” activity was confirmed by the activation of three genes, demonstrating Alla@ZIF8‐Gel's capacity to modulate inflammatory mediators and immune responses. Additionally, two genes were activated under “Antioxidant” function, directly validating the material's unique capacity to mitigate oxidative damage.

### 2.12 Anti‐Inflammatory Effects In Vivo

**FIGURE 9:**
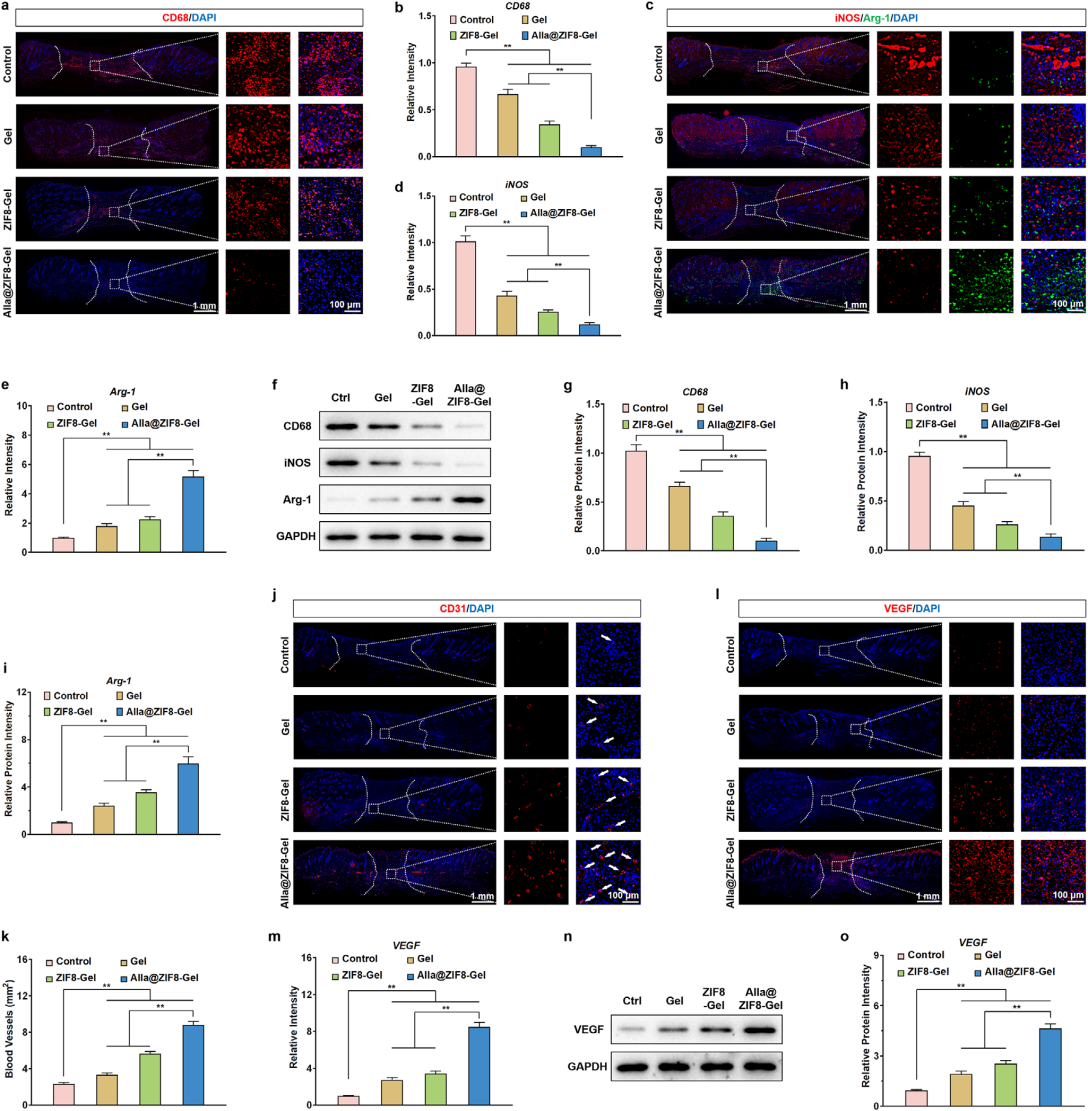
**The in vivo anti‐inflammatory and pro‐angiogenic effects of Alla@ZIF8‐Gel**. (a) Representative CD68 immunofluorescence images of wound tissues in each group of rats after 14 days of corresponding treatment. (b) Quantitative analysis of CD68 relative fluorescence intensity. (c) Representative iNOS/Arg‐1 immunofluorescence images of wound tissues in each group of rats after 14 days of treatment. (d,e) Quantitative analysis of iNOS and Arg‐1 relative fluorescence intensity. (f) Representative Western blotting images of CD68, iNOS, and Arg‐1 protein in wound healing tissue of rats in each group. (g–i) Quantitative analysis of relative protein expression of CD68, iNOS, and Arg‐1. (j) Representative CD31 immunofluorescence images of wound tissues in each group of rats after corresponding treatment. (k) Quantitative analysis of the neovascularization area. (l) Representative VEGF immunofluorescence images of wound tissues in each group of rats after corresponding treatment. (m) Quantitative analysis of VEGF relative fluorescence intensity. (n) Representative Western blotting images of VEGF protein in wound healing tissue of rats in each group. (o) Quantitative analysis of relative protein expression of VEGF. Data are shown as the mean ± SD, *p<0.05 and **p<0.01. Statistical analysis between groups was conducted using One‐way ANOVA.

The mitigation of wound inflammation is of critical importance in the healing of infected wounds [62]. Effective control of excessive inflammatory responses can reduce tissue damage and improve the wound microenvironment [63]. By diminishing the release of pro‐inflammatory mediators, local oedema is alleviated, thereby creating favourable conditions for cell proliferation and tissue regeneration [64]. Furthermore, inflammation resolution facilitates pathogen clearance, accelerates the repair process, minimizes scar formation, and enhances the overall quality of healing. Macrophages, as pivotal immune cells throughout the wound repair process, play a decisive role in orchestrating healing outcomes [65]. CD68, a canonical marker of macrophages, is widely employed to evaluate the inflammatory status of injured tissues. In this study, immunofluorescence staining was utilized to assess CD68 expression in wound tissues to validate the results of tissue transcriptome sequencing. As illustrated in Figure 9a,b, the fluorescence intensity of CD68 was markedly lower in the Gel group compared to the Control group, likely attributable to the moist, occlusive environment provided by the Gel and its inherent antimicrobial properties, which collectively shielded the wound from external bacterial invasion and thereby attenuated inflammation to some extent. In comparison to both the Control and Gel groups, the ZIF8‐Gel group exhibited a further significant reduction in CD68 fluorescence intensity, suggesting that ZIF8‐Gel not only formed a protective barrier but also leveraged ZIF8 nanoparticles to exert bactericidal and pro‐proliferative effects via sustained zinc ion release. Notably, the Alla@ZIF8‐Gel group displayed the lowest CD68 fluorescence intensity among all groups, a result attributed to the synergistic actions of Alla loaded in ZIF8 nanoparticles, which coordinated with quaternary ammonium carboxymethyl chitosan to alleviate oxidative stress, suppress bacterial growth, reduce infection, and mitigate inflammation. To elucidate the underlying mechanism by which Alla@ZIF8‐Gel alleviates wound inflammation, immunofluorescence staining was performed to evaluate the expression of Arg‐1 and iNOS in wound tissues. As shown in Figure 9c–e, Alla@ZIF8‐Gel treatment led to a significant reduction in M1 macrophages and a concomitant increase in M2 macrophages within the wound area compared to the Control group, indicating that Alla@ZIF8‐Gel effectively promotes M2 polarization of macrophages in infected wounds in vivo. This phenomenon is associated with the potent ROS‐scavenging activity of Res and the immunomodulatory function of Alla, consistent with our prior in vitro findings. Collectively, the potent in vivo anti‐inflammatory effect of Alla@ZIF8‐Gel can be ascribed to the synergistic interplay between the hydrogel's robust antimicrobial properties and its capacity to alleviate oxidative stress. WB analysis of CD68, Arg‐1, and iNOS protein expression revealed trends consistent with the immunofluorescence results (Figure 9–i), further corroborating that Alla@ZIF8‐Gel mitigates inflammation in infected wound tissues by promoting M2 macrophage polarization through the clearance of excess ROS.

### 2.13 Promotion of Neovascularization In Vivo

The extent of neovascularization is a critical determinant of wound healing quality, as it ensures adequate oxygen and nutrient supply to support cellular metabolism and tissue regeneration [66]. By establishing a new microvascular network, angiogenesis facilitates the clearance of necrotic debris and metabolic by‐products, thereby improving the local microenvironment and promoting infection control. Furthermore, robust neovascular formation accelerates granulation tissue development and re‐epithelialization, ultimately reducing wound closure time and enhancing healing outcomes [67]. In this study, the density of newly formed blood vessels in healed tissues across experimental groups was assessed on day 14 via CD31 immunofluorescence staining. As shown in Figure 9j,k, the neovascularized area in the Gel and ZIF8‐Gel groups was markedly greater than that in the Control group. Most notably, the Alla@ZIF8‐Gel group exhibited a significantly higher number of new blood vessels at the wound site compared to both the Gel and ZIF8‐Gel groups. This enhanced angiogenic response is likely attributable to the microenvironment provided by Alla@ZIF8‐Gel, which supports cellular growth, while the incorporated Alla@ZIF8 nanoparticles further promote the migration and proliferation of endothelial and epithelial cells. Neovascularization during wound healing is closely associated with growth factors secreted by endothelial cells, including vascular endothelial growth factor (VEGF), epidermal growth factor (EGF), and basic fibroblast growth factor (bFGF). Among these, VEGF serves as the central regulator of angiogenesis, specifically promoting endothelial cell proliferation, migration, and tubulogenesis [68]. By enhancing vascular permeability and activating downstream signaling pathways, VEGF induces vascular sprouting and network formation, thereby accelerating the maturation and stabilization of newly formed vessels. Elevated VEGF expression not only ensures revascularization during tissue repair but also provides essential microenvironmental support for cellular metabolism and immune responses. Immunofluorescence staining of wound sections revealed significantly higher VEGF expression in the Alla@ZIF8‐Gel group compared to the Control, Gel, and ZIF8‐Gel groups (Figure 9l,m). Furthermore, WB analysis confirmed consistent trends in VEGF protein expression, corroborating the immunofluorescence results (Figure 9n,o). These findings indicate that Alla@ZIF8‐Gel synergistically enhances neocapillary formation by promoting endothelial cell migration and proliferation and upregulating the secretion of pro‐angiogenic growth factors within the wound tissue, thereby effectively accelerating the repair of infected wounds. To evaluate the in vivo biocompatibility of Alla@ZIF8‐Gel, histological assessment of major organs (heart, liver, spleen, lung, and kidney) was performed using HE staining. No significant histopathological differences were observed between the Control group and the Gel, ZIF8‐Gel, and Alla@ZIF8‐Gel groups (Figure S20). Additionally, serum levels of alkaline phosphatase (ALP), alanine aminotransferase (ALT), and aspartate aminotransferase (AST) showed no significant variations among the groups (Figure S21), further demonstrating the excellent biocompatibility of Alla@ZIF8‐Gel and supporting its suitability as an antimicrobial wound dressing.

## 3 Conclusion

In this study, we successfully engineered a multifunctional therapeutic system for infected wound management, based on a hydrogel composed of methacrylated quaternized carboxymethyl chitosan integrated with Alla‐loaded ZIF8 nanoparticles (Alla@ZIF8‐Gel). By synergistically combining the bactericidal action of quaternized chitosan, the zinc ion interference from ZIF8, the immunomodulatory properties of Alla, and the antioxidant effects of Res, this system achieves efficient eradication of drug‐resistant bacteria and coordinated modulation of the wound microenvironment. Experimental results demonstrate that Alla@ZIF8‐Gel exhibits bacteriostatic rates exceeding 95% against both *MRSA and E. coli*, significantly outperforming single‐component systems, through mechanisms involving disruption of bacterial membrane integrity and induction of intracellular content leakage. Furthermore, the hydrogel exhibits excellent injectability, robust self‐healing properties, and strong tissue adhesion, enabling it to conform to irregular wound geometries, maintain intimate contact with tissues, and facilitate in situ sealing with sustained drug release. Notably, the combined action of Res and Alla mitigates oxidative damage by scavenging ROS, while playing a pivotal role in immunomodulation. This combination promotes the polarization of macrophages toward the M2 phenotype, markedly suppresses excessive inflammatory responses, downregulates the expression of pro‐inflammatory cytokines TNF‐α and IL‐6, and upregulates anti‐inflammatory factors IL‐10 and Arg‐1. Consequently, by incorporating allantoin‐loaded ZIF8 nanoparticles, our hydrogel achieves intelligent, on‐demand release of pro‐regenerative therapeutics that is synchronized with the wound's inflammatory status. This is complemented by the inherent, broad‐spectrum antibacterial activity of the quaternized chitosan matrix and the potent, sustained ROS‐scavenging capacity of the chemically conjugated resveratrol. The resulting synergistic interplay creates a regenerative microenvironment that effectively manages infection, mitigates inflammation, and promotes tissue repair in a coordinated manner. Therefore, we now position our work not merely as an incremental improvement but as a novel and superior paradigm for next‐generation intelligent wound dressings. This integrated ‘antibacterial‐anti‐inflammatory‐antioxidant‐immunomodulatory’ design transcends the mechanistic limitations of conventional materials and offers a novel strategy for the precise treatment of chronic infected wounds, highlighting the broad translational potential of multifunctional biomaterials in clinical applications.

## 4 Experimental Section

### 4.1 Materials

Carboxymethyl chitosan (CC, carboxymethylation ≥ 85%), glycidyl methacrylate (GMA), Glycidyltrimethylammonium chloride (GTMAC), resveratrol (Res), 2‐methylimidazole (2‐MI), allantoin (Alla), 1‐(3‐dimethylaminopropyl)‐3‐ethylcarbodiimide hydrochloride (EDC), 4‐dimethylaminopyridine (DMAP), dimethyl sulfoxide (DMSO), lithium phenyl (2,4,6‐trimethylbenzoyl) phosphinate (LAP) were purchased from Adamas Reagent Co. Ltd (Shanghai, China). Zinc acetate dihydrate (Zn(OAc)_2_·2H_2_O), sodium hydroxide (NaOH), and absolute ethyl alcohol were provided by Sinopharm Chemical Reagent Co., Ltd. (Shanghai, China). All chemicals were of analytical grade without further purification.

### 4.2 Preparation of Alla@ZIF8 nanoparticles

First, 5 mmol Zn(OAc)_2_·2H_2_O and 50 mmol 2‐MI were sequentially dissolved in 70 mL of deionized water under magnetic stirring, and then Alla was added (5 mg/mL) and stirred for 30 min. Subsequently, the mixed solution was transferred to a 100 mL Teflon‐lined stainless steel autoclave and heated at 120°C for 24 h. After cooling to room temperature, the resulting sediments were centrifugally collected at 6000 rpm and rinsed with deionized water and absolute ethyl alcohol three times, respectively. Finally, the Alla@ZIF8 powders were obtained after in vacuum‐dried for 12 h at 60°C. For the preparation of ZIF8 nanoparticles, the procedures were the same as the Alla@ZIF8 nanoparticles, except without the addition of Alla.

### 4.3 Synthesis of RMQCC

Firstly, 8 g of CC powders were dissolved in deionized water (2% w/v) with magnetic stirring, followed by the GMA (4 mL) and GTMAC (0, 2, 4, and 8 g) were simultaneously added into the CC solution. Afterwards, the pH value of the mixed solution was adjusted to 9 with NaOH solution (1 M) and reacted for 1 h at 60°C under magnetic stirring. After the reaction was completed, the mixed solution was dialyzed against deionized water using the dialysis tubing with a molecular weight cutoff of 8–14 kDa (VWR Scientific, USA) for 5 days at room temperature to remove unreacted GMA, GTMAC, and NaOH, and the purified solution was frozen overnight at −80°C and lyophilized to obtain the MQCC. For the synthesis of RMQCC, the lyophilized MQCC was dissolved in the mixed solution of DMSO/H_2_O (1:1 in vol) at a concentration of 2% w/v, followed by the DMAP and EDC (1:10 in molar) were added together. Subsequently, Res was added to the MQCC solution and reacted for 24 h at room temperature. After the reaction was completed, the mixed solution was successively dialyzed against a 50% DMSO solution and deionized water using the dialysis tubing with a molecular weight cutoff of 8∼14 kDa for 3 days at room temperature. Finally, the purified solution was frozen overnight at −80°C and lyophilized to obtain the RMQCC. According to the additive amount of GTMAC aforementioned, the obtained RMQCC were named as RMQCC0, RMQCC2, RMQCC4, and RMQCC8.

### 4.4 Synthesis of Alla@ZIF8‐Gel nanocomposite hydrogel

The Alla@ZIF8 nanoparticles were dispersed into deionized water at a desired concentration, and then the lyophilized RMQCC were added into the Alla@ZIF8 dispersion at a concentration of 2% w/v, followed by the addition of the LAP photoinitiator (0.2% w/v) until complete dissolution. With the mixed solution exposed to 405 nm UV light (∼5 s), the Alla@ZIF8‐Gel nanocomposite hydrogel was obtained. By using ZIF8 dispersion and deionized water instead of Alla@ZIF8 dispersion, the ZIF8‐Gel and Gel hydrogels were obtained, respectively.

### 4.5 Characterization of nanoparticles and hydrogels

The chemical structure of the RMQCC was analyzed by 1H nuclear magnetic resonance (^1^H NMR, Bruker AV6400, 600 MHz, Bruker, Germany) with D_2_O (4.74 ppm) as reference. The micromorphology of the nanoparticles and hydrogels was investigated by field‐emission scanning electron microscope (FE‐SEM, Apreo S, Thermo Scientific, USA). For the hydrogel, the samples were freeze‐dried, and liquid‐nitrogen quenched, and then the cross‐section of the fractured hydrogels were sputtered with a thin layer of gold before the SEM observation. The particle size of the nanoparticles and pore size of the hydrogel were calculated based on the statistical results of 20 particles/pores from SEM images using ImageJ software. The micromorphology and element mapping of the nanoparticles were further investigated with a transmission electron microscope (TEM, Talos F200X, Thermo Scientific, USA). All the optical photographs and videos were taken by a smartphone (Huawei Mate 50, China). Surface potential of the nanoparticles was measured with a zeta potential analyzer (Zetasizer Nano ZSE, Malvern Instruments, England) at pH 7.0. Thermogravimetric analysis (TGA) of the nanoparticles was performed by a thermal gravimetric analyzer (Q50, TA Instruments, USA) at a heating rate of 5°C/min from 30°C to 800°C under a nitrogen atmosphere. Phase composition of the nanoparticles was characterized with an X‐ray diffractometer (XRD, D8A25, Bruker, Germany) using Cu Ka radiation source (*k* = 1.54 Å) at 35 kV in the 2*θ* range of 10°–80° with a scan rate of 5°/min. Fourier transform infrared (FT‐IR) spectra of the nanoparticles and hydrogels were recorded by an infrared spectrometer (Vertex 70, Bruker, Germany) with the range from 400 to 4000 cm^−1^ at room temperature, and ultraviolet–visible (UV–vis) absorption spectra of the nanoparticles was recorded by an ultraviolet‐visible spectrophotometer (Evolution 350, Thermo Scientific, USA) with a wavelength range of 200–800 nm at room temperature.

### 4.6 Ion and drug release studies

To understand the hydrogels’ Zn^2+^ ion release behavior, 100 mg of hydrogel samples was immersed in 10 mL of PBS solution with different pH value (5.0, 6.5, and 7.4) at 37°C, and 5 mL of immersion solution was withdrawn at pre‐determined time points (6, 12, 24, 48, 72, 96 and 120 h) for concentration analysis by ICP‐OES an inductively coupled plasma optical emission spectrometry (ICP‐OES, iCAP PRO, Thermo Scientific, USA). After this, the residual immersion solution was replenished with the same volume of fresh PBS. The Alla loading content in Alla@ZIF8 nanoparticles was determined by subtracting the amount of unentrapped Alla in the supernatant from the initial total amount of Alla. Here, the supernatant was obtained by centrifuging Alla@ZIF8 dispersion at 8000 rpm for 10 min, and the unentrapped Alla was quantified by UV–vis spectrophotometer at λ = 215 nm. To investigate the Alla release, 100 mg of hydrogel samples were immersed in 10 mL of PBS solution with different pH value (5.0, 6.5, and 7.4) at 37°C and 5 mL of immersion solution was withdrawn at pre‐determined time points (6, 12, 24, 48, 72, 96, and 120 h). After being centrifuged at 8000 rpm for 10 min, the cumulative percentage of released Alla in the supernatants was quantified using a UV–Vis spectrophotometer at 215 nm by dividing the amount of Alla released at each time point by the total amount of Alla in the initial hydrogel. A calibration curve was prepared for the quantification of Alla release.

### 4.7 Swelling, water retention, and degradation studies

To measure the swelling ratio of the hydrogels, the appropriate amount of the dried hydrogel (W_1_) was immersed in PBS solution (pH = 7.4) at 37°C. At each pre‐determined time point, the weight of the swelled hydrogel (W_2_) was taken out and weighed after blotting the excess water on the surface of the hydrogel gently on a filter paper. The swelling ratio was then calculated by the formula:

(1) $$\mathrm{Swelling}\;\mathrm{ratio}\left(\%\right)=\left[\left(W_{2}-W_{1}\right)/W_{1}\right]\times 100\%$$

The water retention capacity of the hydrogels was measured as follows: the appropriate amount of the dried hydrogel (W_1_) was immersed in PBS solution (pH = 7.4) at 37°C for 24 h to achieve full swelling (W_s_), and then the swelled hydrogel was separately placed in empty beakers at room temperature. At each pre‐determined time point, the weight of the resulting hydrogel was recorded as W_2_. The water retention ratio was calculated according to the formula:

(2) $$\mathrm{Water}\;\mathrm{retention}\;\mathrm{ratio}\left(\%\right)=\left[\left(W_{2}-W_{1}\right)/\left(W_{s}-W_{1}\right)\right]\times 100\%$$

To evaluate the degradation behavior of the hydrogels, the appropriate amount of dried hydrogel (W_1_) was immersed into PBS solution (pH = 7.4) at 37°C. After each pre‐determined time interval, the hydrogel was taken out, lyophilized and weighed (W_2_). The degradation ratio was calculated using the formula:

(3) $$\mathrm{Degradation}\;\mathrm{ratio}\left(\%\right)=\left[\left(W_{1}-W_{2}\right)/W_{1}\right]\times 100\%$$

### 4.8 ROS‐scavenging ability in vitro

The scavenging ability of hydrogels against hydroxyl radical (·OH) was determined according to the previously reported method [69]. First, 300 µL of H_2_O_2_ was added to 2 mL of mixed solution containing 5 mM FeSO_4_·7H_2_O and 5 mM salicylic acid to initiate the reaction for 20 min, followed by 300 µL of hydrogel was added. After being incubated in dark for 30 min at 37°C, the absorbance of the mixed solution was measured at 510 nm. The ·OH scavenging ability of hydrogels was calculated by the formula: Scavenging ratio = [(A_0_−A_1_)/(A_0_)] × 100%, where A_0_ and A_1_ represent the absorbance of the solution in the absence and presence of hydrogel. To examine the scavenging ability of hydrogels against superoxide radical (O_2_^·−^), 2 mL of pyrogallol (3 mM) was added to 12 mL of Tris‐HCl (50 mM, pH 8.1) and stood for 5 min in the dark to generate the O_2_^·−^, followed by 300 µL of hydrogel was added. After being incubated in the dark for 30 min at 37°C, the absorbance of the mixed solution was measured at 299 nm. The O_2_^·−^ scavenging ability of hydrogels was calculated by the same formula as ·OH. To evaluate the DPPH free radical (ABTS·) scavenging ability of the hydrogels, 300 µL of hydrogel was added to 1 mL of DPPH ethanol solution (0.1 mM) and then stored in the dark for 30 min. The absorbance of the solution was measured at 517 nm, and the DPPH· scavenging ability of hydrogels was calculated by the same formula as ·OH. A commercial total antioxidant capacity (T‐AOC) assay kit was adopted to measure ABTS free radical (ABTS·) scavenging ability of the hydrogels. After the ABTS· working solution was prepared following the operating instructions, 300 µL of hydrogel was added and then incubated in the dark for 6 min. Subsequently, the absorbance of the solution was measured at 734 nm, and the DPPH· scavenging ability of hydrogels was calculated by the same formula as OH.

### 4.9 Rheological measurement

The rheological properties of the hydrogels were measured using a rotary rheometer (DHR‐2, TA Instruments, USA). A cylindrical hydrogel (25 mm in diameter, 2 mm in thickness) was placed between two 25 mm parallel plates at 37°C. The frequency sweep test was performed with a frequency scanning range of 0.1–100 rad/s at 1.0% strain, and the strain sweep test was performed with the strain range from 0.1% to 1000% at 1 rad/s. The shear‐thinning property of the hydrogels was assessed by measuring the linear viscosity with shear rate varying from 0.01 to 100 s^−1^ at 37°C. For the alternate step strain sweep test, the oscillatory strain was switched between a low strain of 1.0% and a high strain of 400% with a constant frequency of 1 rad/s, ensuring a duration of 60 s for each strain value.

### 4.10 Mechanical property test

The mechanical properties of the hydrogels were tested by a universal material testing machine (Instron 5982, Instron, USA) equipped with a 100 N load cell. For the tensile test, the rectangular hydrogel samples (size: 30 × 6 × 1 mm) were clamped on the machine, and the test was performed at a speed of 2 mm/min. For compression test, the cylindrical hydrogel samples (size: 25 × 20 mm) were mounted on the machine, and the test was carried out with a speed of 2 mm/min at a maximum strain of 80%. All these tests were repeated six times.

### 4.11 Adhesive property test

The adhesive capacity of the hydrogels was evaluated by the lap‐shear test and macroscopic observation. In the lap‐shear test, 200 µL of the hydrogel precursor solution was uniformly coated onto a fresh porcine skin (50 × 10 mm), and then was photo‐crosslinked under 405 nm UV light. Whereafter, another fresh porcine skin with the same was instantly covered on the hydrogel containing porcine skin and then let stand for 2 h at 37°C. The contact area between two porcine skins was 10 × 10 mm^2^. The adhesive property was tested using a universal material testing machine (Instron 5982, Instron, USA) equipped with a 100 N load cell at a speed of 2 mm/min. The adhesion strength is calculated with the maximum force in the force‐displacement curve divided by the contact area [70]. All these tests were repeated 6 times. To macroscopically observe the adhesive capacity of the hydrogel, the hydrogel was prepared and adhered to the surface of different materials, including rubber, glass, ceramic, PTFE, metal, and plastic, and then the corresponding adhesion photographs were shot.

### 4.12 Hemostatic ability test

The hemostatic potential of the hydrogels was assessed by a rat liver hemorrhage model. Briefly, a bleeding lesion was created by puncturing the liver with a 14G needle, and then the hydrogel was promptly injected into the bleeding area for observation. Documentation of hemostasis was achieved through photography following complete cessation of bleeding. The control group consisted of rats with liver hemorrhage that did not receive any treatment.

### 4.13 Self‐healing behavior study

To evaluate the self‐healing ability of the hydrogels, the rhodamine B and riboflavin phosphate sodium colored cylindrical hydrogels were equally sectioned into semi‐cylindrical hydrogels, respectively, and then the different colored semi‐circular hydrogels were contacted for 1 h at 37°C.

### 4.14 Biocompatibility evaluation in vitro

NIH‐3T3 and RAW 264.7 cells were seeded separately into 96‐well plates and co‐cultured with the respective hydrogels. Following a 3‐day incubation period, cell viability was assessed using a Live/Dead staining kit according to the manufacturer's instructions, and the cells were subsequently imaged using confocal microscopy. Furthermore, apoptosis in both NIH‐3T3 and RAW 264.7 cells across all experimental groups was evaluated via a TUNEL staining assay. The effect of the hydrogels on cell proliferation was determined using an EdU staining kit, with the results documented by confocal microscopy. Subsequently, the proliferation of NIH‐3T3 cells, following co‐culture with the hydrogels for 3 and 7 days at 37°C in a 5% CO_2_ atmosphere, was quantified using a CCK‐8 assay. Finally, the influence of the hydrogels on cell adhesion was investigated by staining with rhodamine‐conjugated phalloidin.

### 4.15 Hemolytic activity assessment

The hemocompatibility of the Alla@ZIF8‐Gel was evaluated through a hemolysis assay. Initially, erythrocytes were isolated from rat peripheral blood by centrifugation at 1000 rpm for 10 min. The resulting erythrocytes were diluted with PBS to a final concentration of 5% (v/v) for subsequent experiments. The erythrocyte suspension was mixed with each hydrogel and added to 24‐well plates, followed by co‐incubation at 37°C for 2 h. The resulting mixture was then centrifuged for 10 min, and the supernatant (100 µL) was transferred to a 96‐well plate. The absorbance of each sample at 540 nm was measured using a microplate reader. Triton X‐100 solution and PBS buffer served as the positive and negative controls, respectively. The hemolysis rate was calculated using the formula:

(4) $$Hemolysis\left(\%\right)=\left[\left(A_{e}-A_{n}\right)/\left(A_{p}-A_{n}\right)\right]\times 100\%$$

where A_e_ represents the absorbance of the experimental group, A_p_ that of the Triton X‐100 positive control, and A_n_ that of the PBS negative control.

### 4.16 Scratch assay

The pro‐migratory effect of Alla@ZIF8‐Gel on NIH‐3T3 and RAW 264.7 cells was evaluated using a scratch wound assay. NIH‐3T3 cells were first trypsinized into a single‐cell suspension and co‐cultured with each hydrogel in 6‐well plates. Upon reaching near‐confluence (∼100% density), a linear scratch was introduced using a sterile pipette tip, ensuring consistent pressure and maintaining a vertical orientation throughout the procedure. Subsequently, the culture medium was aspirated, and detached cells were removed by washing with PBS. Fresh low‐serum medium was then added, and the plates were incubated at 37°C in a 5% CO_2_ atmosphere for 24 h. Cell migration was monitored, and images of the scratch were captured at designated time points to assess wound closure. The same experimental protocol was applied to evaluate the effect of Alla@ZIF8‐Gel on the migration of RAW 264.7 cells.

### 4.17 Antioxidant damage capacity test in vitro

To assess the antioxidant capacity of Alla@ZIF8‐Gel under high oxidative stress conditions (0.1 mM H_2_O_2_), NIH‐3T3 cells were co‐cultured with the hydrogel for 24 h, followed by evaluation of intracellular ROS levels using DCFH‐DA and DHE probes. Concurrently, flow cytometry was employed to quantify intracellular ROS levels, enabling a robust assessment of the hydrogel's antioxidant efficacy. The impact of each hydrogel on apoptosis in NIH‐3T3 and RAW 264.7 cells under high oxidative stress was evaluated using a TUNEL apoptosis detection kit. Briefly, NIH‐3T3 and RAW 264.7 cells were fixed with 4% paraformaldehyde and permeabilized with Triton X‐100. Subsequently, TUNEL reaction mixture was applied and incubated for 1 h. Following nuclear counterstaining with DAPI, fluorescence images were captured using confocal microscopy to visualize apoptotic cells.

### 4.18 Macrophage polarization evaluation in vitro

Following co‐culture of macrophages with each hydrogel, the expression of M1 and M2 subtype‐associated marker genes, proteins, and inflammatory cytokines was assessed using RT‐qPCR, immunofluorescence staining, flow cytometry, Western blotting, and ELISA. Initially, mRNA expression levels of iNOS, TNF‐α, IL‐6, Arg‐1, IL‐10, and TGF‐β were quantified. Primer sequences for these targets are provided in Table S2. RT‐qPCR was performed under the following cycling conditions: 95°C for 30 s, followed by 40 cycles of 95°C for 15 s and 60°C for 1 min. Fluorescence data were subsequently analyzed for quantitative determination of gene expression. Immunofluorescence staining was employed to evaluate the expression of iNOS, Arg‐1, CD86, and CD206 in macrophages from each group. Briefly, after co‐culture with the hydrogels for 3 days, cells were fixed with paraformaldehyde for 20 min and washed three times with PBS. Permeabilization was performed using 0.3% Triton X‐100, followed by blocking with BSA solution for 60 min. Cells were then incubated overnight at 4°C with primary antibodies, washed three times with PBS, and incubated with secondary antibodies for 2 h at room temperature. After DAPI counterstaining for 5 min, fluorescence images were acquired using confocal microscopy. Immunofluorescence intensity was quantitatively analyzed using ImageJ software. In addition, flow cytometry was utilized to determine the percentages of CD86‐positive and CD206‐positive macrophages in each group. The secretion of cytokines by macrophages was measured using ELISA kits. After centrifugation of the cell culture medium, supernatants were transferred to 96‐well plates. According to the manufacturer's instructions, HRP‐conjugated detection antibodies were added sequentially. Following incubation and washing, tetramethylbenzidine (TMB) substrate was applied for color development. Absorbance was measured at 450 nm using a microplate reader, and cytokine concentrations were determined by interpolation from a standard curve.

### 4.19 Antibacterial test in vitro

The in vitro antibacterial activity of each hydrogel was assessed through co‐culture with bacterial strains. Escherichia coli (E. coli, ATCC 25922) and methicillin‐resistant Staphylococcus aureus (MRSA, ATCC 33591) were employed as representative Gram‐negative and Gram‐positive bacteria, respectively. Hydrogels were uniformly distributed into the wells of a 24‐well plate, after which bacterial suspensions (∼10^6^ CFU/mL) were applied to their surfaces for a 24‐h co‐culture. The Control group received no treatment. Following incubation, bacterial suspensions were collected from each hydrogel surface, diluted 100‐fold in PBS, and plated onto agar plates. After 24 h of incubation at 37°C, images of the agar plates were captured, and colony‐forming units (CFU) were enumerated. In addition, the bactericidal efficacy of each hydrogel was evaluated using a SYTO‐9/PI bacterial viability assay. Bacterial suspensions from each treatment group were incubated with the working solution for 10 min, during which live bacteria emitted green fluorescence and dead bacteria emitted red fluorescence. Bacterial viability was documented using confocal laser scanning microscopy. Furthermore, SEM was employed to examine morphological changes in bacteria following 24‐h co‐culture with each hydrogel. Briefly, bacterial samples were fixed, dehydrated, dried, and sputter‐coated with gold before SEM imaging at appropriate magnifications. The bacterial suspensions were then filtered, and the concentration of proteins released from necrotic and lysed bacteria was quantified using a BCA protein assay kit. Additionally, intracellular potassium ion content in bacteria from each group was measured using inductively coupled plasma mass spectrometry.

### 4.20 Infected wound healing assessment in vivo

This study was approved by the Experimental Animal Ethics Committee of the Medical College of Jiaxing University (Approval No. JUMC2025‐148). All animal procedures were performed in accordance with the guidelines for the care and use of laboratory animals established by the institution and the Chinese government. In this study, *MRSA* was selected as the pathogen to establish an infected wound model. Sixty Sprague–Dawley (SD) rats were anesthetized using a gas anesthesia machine. Following depilation, the dorsal skin was disinfected with povidone‐iodine. A full‐thickness circular wound approximately 1 cm in diameter was created on the dorsum using a skin punch biopsy, extending to the muscle layer. Subsequently, 100 µL of an *MRSA* bacterial suspension (∼10^8^ CFU/mL) was inoculated into each wound. Successful wound infection was confirmed 24 h post‐inoculation by agar plate culture, after which the rats were randomly allocated into four groups (*n* = 15 per group): (1) Control group: no treatment; (2) Gel group: treated with Gel; (3) ZIF8‐Gel group: treated with ZIF8‐Gel; (4) Alla@ZIF8‐Gel group: treated with Alla@ZIF8‐Gel. Wounds in each group were rinsed with saline before treatment, and sterile dressings were applied post‐treatment. Wound healing was monitored and photographed on days ‐1, 0, 3, 7, and 14. Wound area was measured using ImageJ software, and the wound closure rate was calculated as follows:

(5) $$\begin{array}{ccc} & & \mathrm{Wound}\;\mathrm{closure}\;\mathrm{rate}\left(\%\right)=\\ & & \;[\left(\mathrm{Initial}\;\mathrm{wound}\;\mathrm{area}-\mathrm{Wound}\;\mathrm{area}\;\mathrm{on}\;\mathrm{observation}\;\mathrm{day}\right)/\\ & & \;\mathrm{Initial}\;\mathrm{wound}\;\mathrm{area}]\times 100\% \end{array}$$

### 4.21 Transcriptome sequencing analysis

Total RNA was extracted from rat wound tissues using Trizol reagent according to the manufacturer's instructions. RNA purity and concentration were assessed using a NanoDrop 2000 spectrophotometer, and RNA integrity was evaluated with an Agilent 2100 Bioanalyzer. Transcriptome sequencing and subsequent bioinformatic analysis were performed by Beijing Novogene Bioinformatics Technology Co., Ltd. In brief, RNA sequencing was performed on the Illumina NovaSeq 6000 platform. Differentially expressed genes were identified using DESeq2, with thresholds set at |log2 fold change| ≥ 1 and adjusted *p* value (padj) < 0.05. Gene Ontology (GO) and Kyoto Encyclopedia of Genes and Genomes (KEGG) enrichment analyses were conducted using ClusterProfiler, while Gene Set Enrichment Analysis (GSEA) was performed with the fgsea package. Gene identifiers were converted from ENSEMBL format to gene symbols, establishing a comprehensive gene annotation mapping system.

### 4.22 Histological analysis

After 14 days of treatment, wound tissues from each group were harvested and fixed in formalin. Paraffin‐embedded sections of the wound tissues were prepared using a cryostat. Following deparaffinization and rehydration, sections were subjected to haematoxylin and eosin (HE) staining to evaluate tissue regeneration, inflammatory cell infiltration, and granulation tissue formation under light microscopy. Concurrently, Masson's trichrome staining was performed to assess collagen deposition, with blue‐stained areas indicating collagen fibres. Immunofluorescence staining for Col‐I, Col‐III, CD68, Arg‐1, iNOS, CD31, and VEGF was conducted to evaluate collagen deposition, inflammation, macrophage polarization, and neovascularization within the wound tissues of each group.

### 4.23 Western blotting analysis

Total protein was extracted from cells or tissues using RIPA lysis buffer, and protein concentration was quantified with a BCA protein assay kit. Subsequently, SDS‐PAGE gels were prepared according to the kit instructions, and protein samples were loaded and separated by electrophoresis. Proteins were then transferred to a PVDF membrane via wet transfer. The membrane was blocked with 5% non‐fat milk for 1 h at room temperature. Primary antibodies, diluted to appropriate concentrations in antibody dilution buffer, were incubated with the membrane overnight at 4°C. Following three washes with TBST buffer, the membrane was incubated with the corresponding secondary antibodies for 2 h at room temperature. After three additional TBST washes, the membrane was treated with enhanced chemiluminescence (ECL) substrate and visualized using a chemiluminescence imaging system. The grayscale values of the protein bands were quantified using ImageJ software.

### 4.24 Statistical analysis

All data were analyzed with GraphPad Prism 8 (GraphPad Software Inc., La Jolla, CA, USA). Experimental results were shown with mean ± SD. One‐way ANOVA and two‐way ANOVA were performed for comparison analysis. Significance levels were set at * *p*<0.05 and ** *p*<0.01.

## Conflicts of Interest

The authors declare no conflicts of interest.

## Data and Materials Availability

All data needed to evaluate the conclusions in the paper are present in the paper and/or the Supplementary Materials. Additional data related to this paper may be requested from the authors.

## Supporting information




**Supporting file**: advs73537‐sup‐0001‐SuppMat.docx.
