# Supplementary material for: Self‐Adaptive Allantoin@ZIF8 Nanocomposite Hydrogel with Resveratrol Synergy for MRSA‐Infected Wound Regeneration
Source: Adv Sci (Weinh). 2026 Mar 16;13(21):e20614. doi: 10.1002/advs.202520614 (PMC13073323; doi:10.1002/advs.202520614)
Supplement: Supplementary file 1 — Supporting file: advs73537‐sup‐0001‐SuppMat.docx. [file ADVS-13-e20614-s001.docx]

**Supporting Information**

**Self-Adaptive Allantoin@ZIF8 Nanocomposite Hydrogel with Resveratrol Synergy for MRSA-Infected Wound Regeneration**

Yongjie Zhu^1,^ **^#^**, Dun Liu^1, #^, Mengran Zhao^2, #^, Xinyi Jian^2^, Enuo Peng^3^, Xingping Zhao^3,^ *, Zezhang Zhu^1,^ *, Bolin Tang ^2, 4, 5,^ *, Benlong Shi^1,^ *

1. Division of Spine Surgery, Department of Orthopedic Surgery, Nanjing Drum Tower Hospital, Affiliated Hospital of Medical School, Nanjing University, Nanjing, 210008, China.
2. School of Materials and Textile Engineering, Jiaxing University, Jiaxing, 314001, China.
3. Department of Gynecology, Third Xiangya Hospital, Central South University, Changsha, 410013, China.
4. Nanotechnology Research Institute, G60 STI Valley Industry & Innovation Institute, Jiaxing University, Jiaxing, 314001, China.
5. Zhejiang Key Laboratory of Bio-based Health Functional Fiber Materials, Jiaxing University, Jiaxing 314001, China.


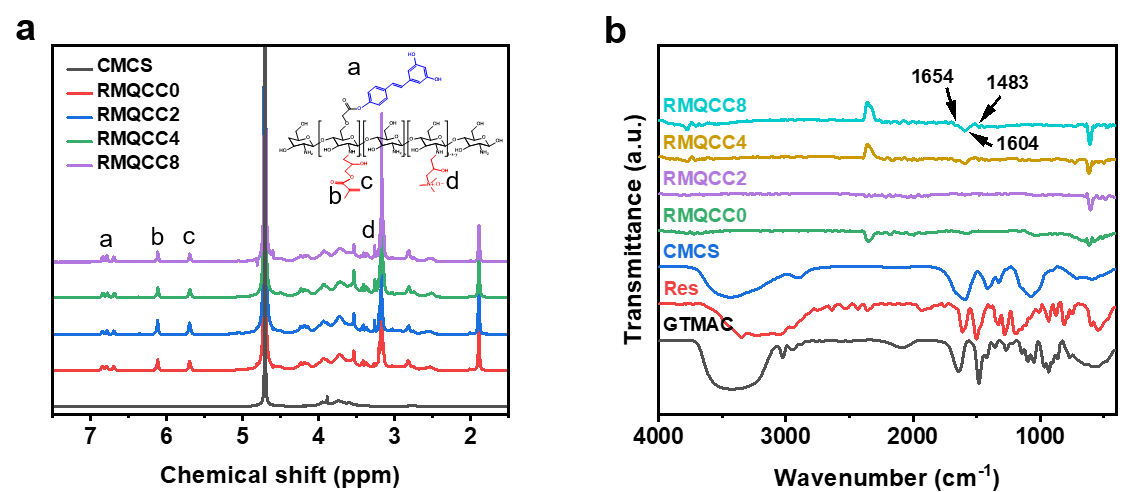


**Figure S1.**  (a) ^1^H NMR spectra of CMCS and RMQCC hydrogels. (b) FTIR spectra of CMCS and RMQCC hydrogels.


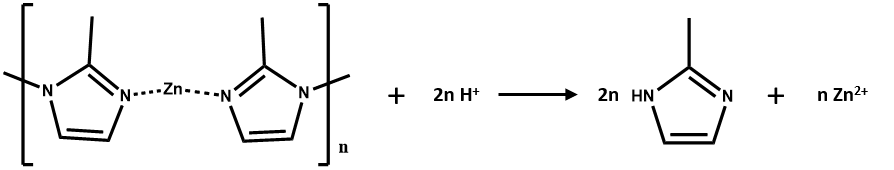


**Figure S2.** Degradation mechanism of ZIF-8 nanoparticles.

**
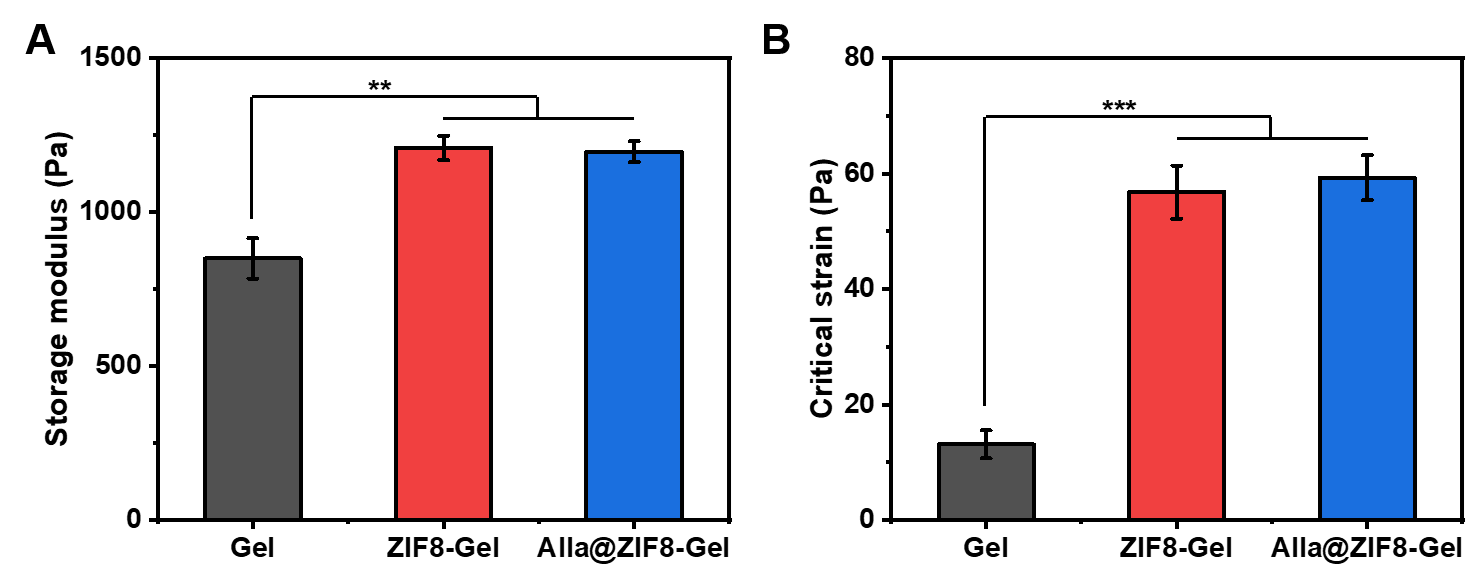
**

**Figure S3.** (a) Quantitative analysis of storage modulus of each hydrogel. (b) Quantitative analysis of Critical strain of each hydrogel. Data are shown as the mean ± SD, *p<0.05 and **p<0.01. Statistical analysis between groups was conducted using One-way ANOVA.

**
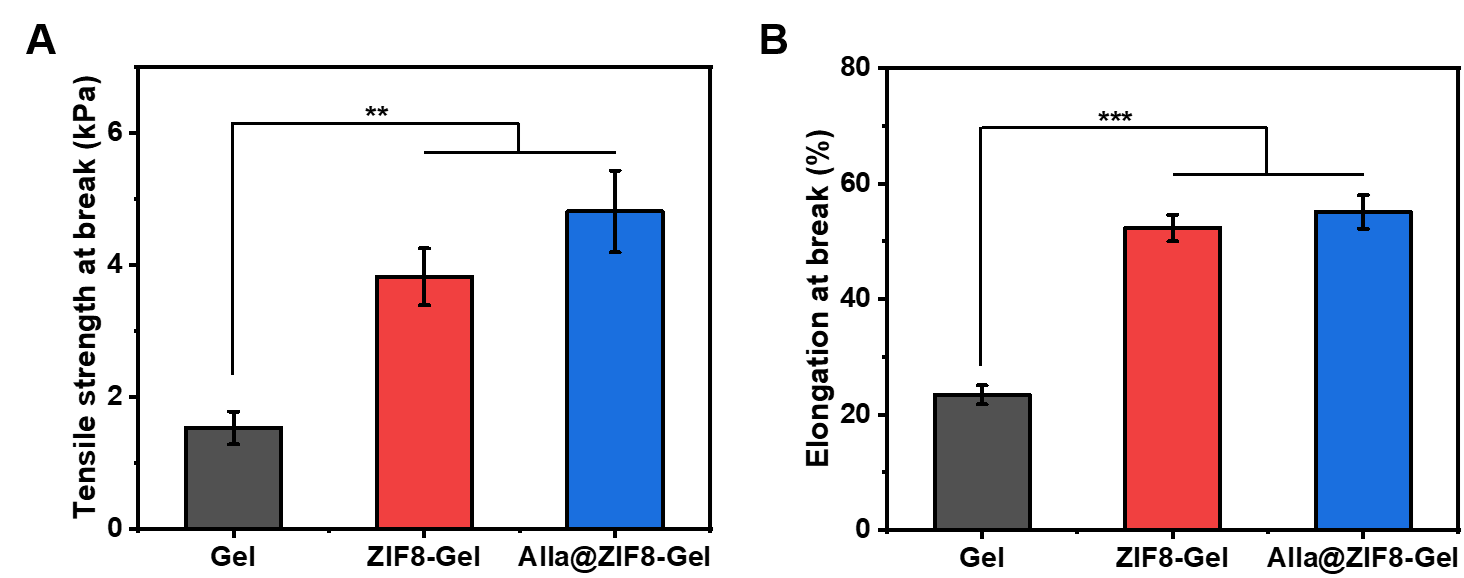
**

**Figure S4.** (a) Quantitative analysis of tensile strength at break of each hydrogel. (b) Quantitative analysis of elongation at break of each hydrogel. Data are shown as the mean ± SD, *p<0.05 and **p<0.01. Statistical analysis between groups was conducted using One-way ANOVA.


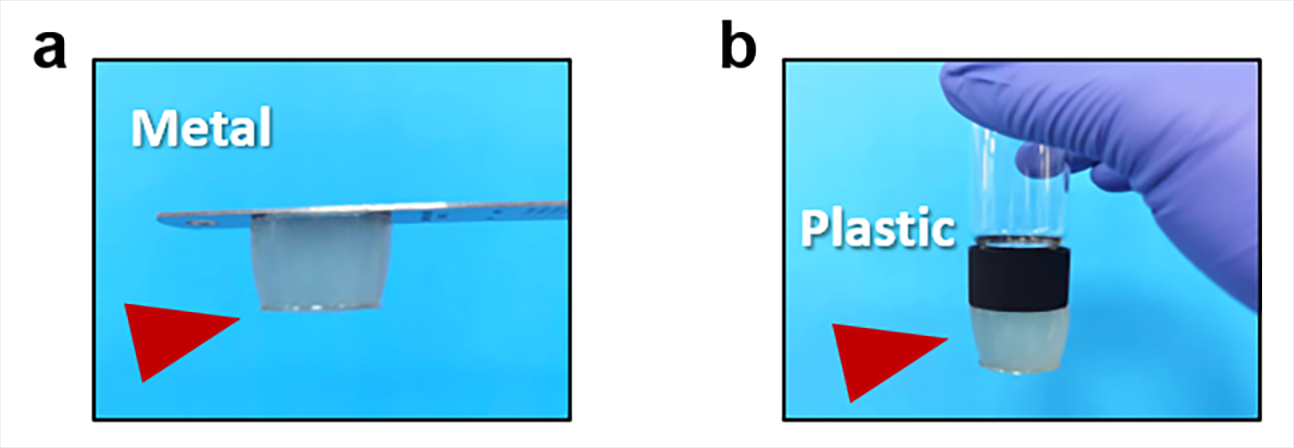


**Figure S5.**  (a) Representative photographs of adhesion of Alla@ZIF8-Gel to metal. (b) Representative photographs of adhesion of Alla@ZIF8-Gel to plastic.

**
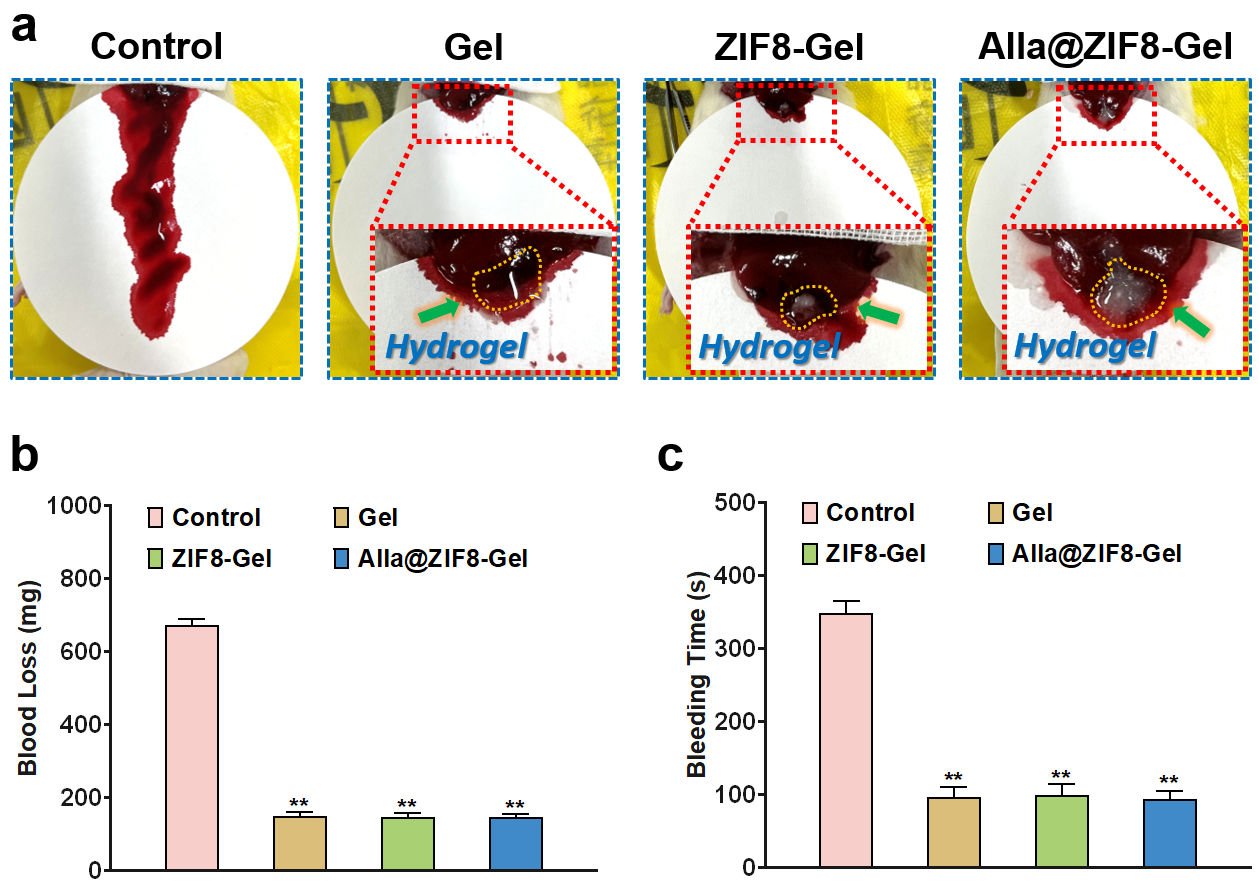
**

**Figure S6.**  (a) Representative photos of liver bleeding lesions treated with different hydrogels. In the yellow dotted box was the implanted hydrogel. (b) The amount of blood loss in the liver of rats after treatment with different hydrogels. (c) The total time of liver bleeding after different treatments. Data are shown as the mean ± SD, *p<0.05 and **p<0.01. Statistical analysis between groups was conducted using One-way ANOVA.


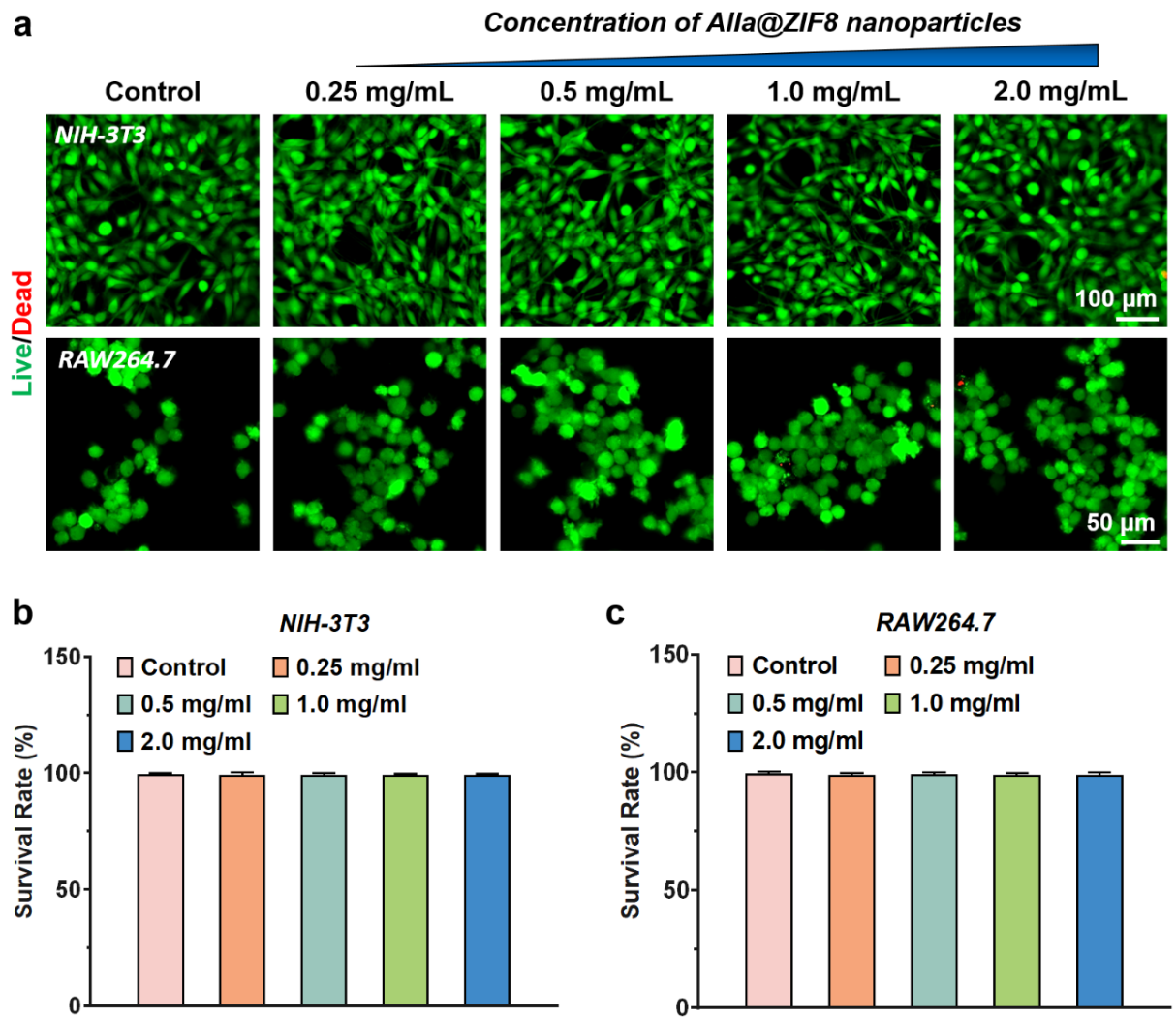


**Figure S7.** Representative Live/Dead staining fluorescence images of NIH-3T3 and RAW 264.7 cells treated with Alla@ZIF8-Gel with different content of Alla@ZIF8 nanoparticles. (b) Quantitative analysis of the proportion of live and dead NIH-3T3 cells. (c) Quantitative analysis of the proportion of live and dead RAW 264.7 cells. Data are shown as the mean ± SD, *p<0.05 and **p<0.01.


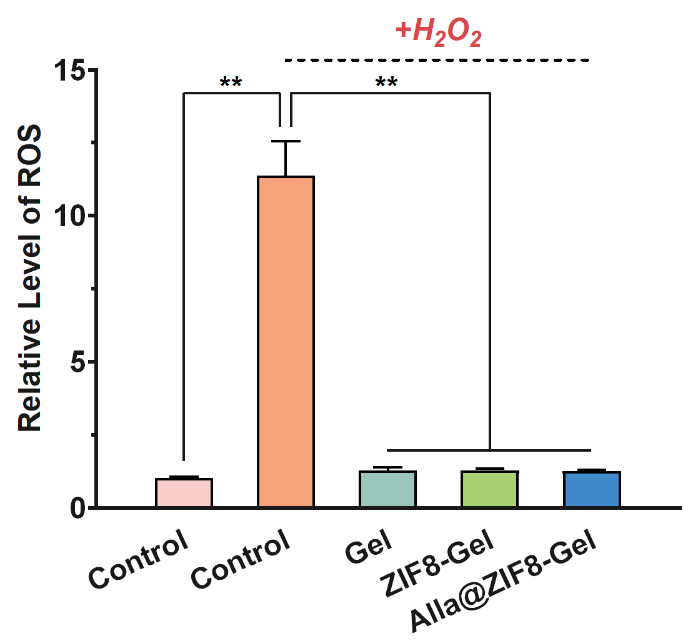


**Figure S8.** Quantitative analysis of intracellular ROS level in various groups of NIH-3T3 cells detected by flow cytometry. Data are shown as the mean ± SD, *p<0.05 and **p<0.01. Statistical analysis between groups was conducted using One-way ANOVA.


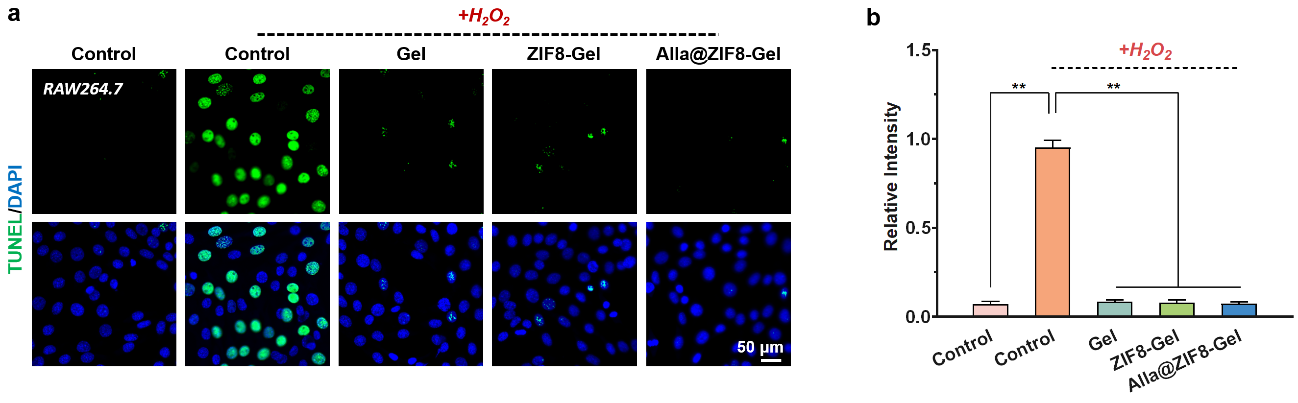


**Figure S9.** (a) Representative TUNEL staining fluorescence images of RAW 264.7 cells after corresponding treatment. (b) Quantitative analysis of relative fluorescence intensity. Data are shown as the mean ± SD, *p<0.05 and **p<0.01. Statistical analysis between groups was conducted using One-way ANOVA.


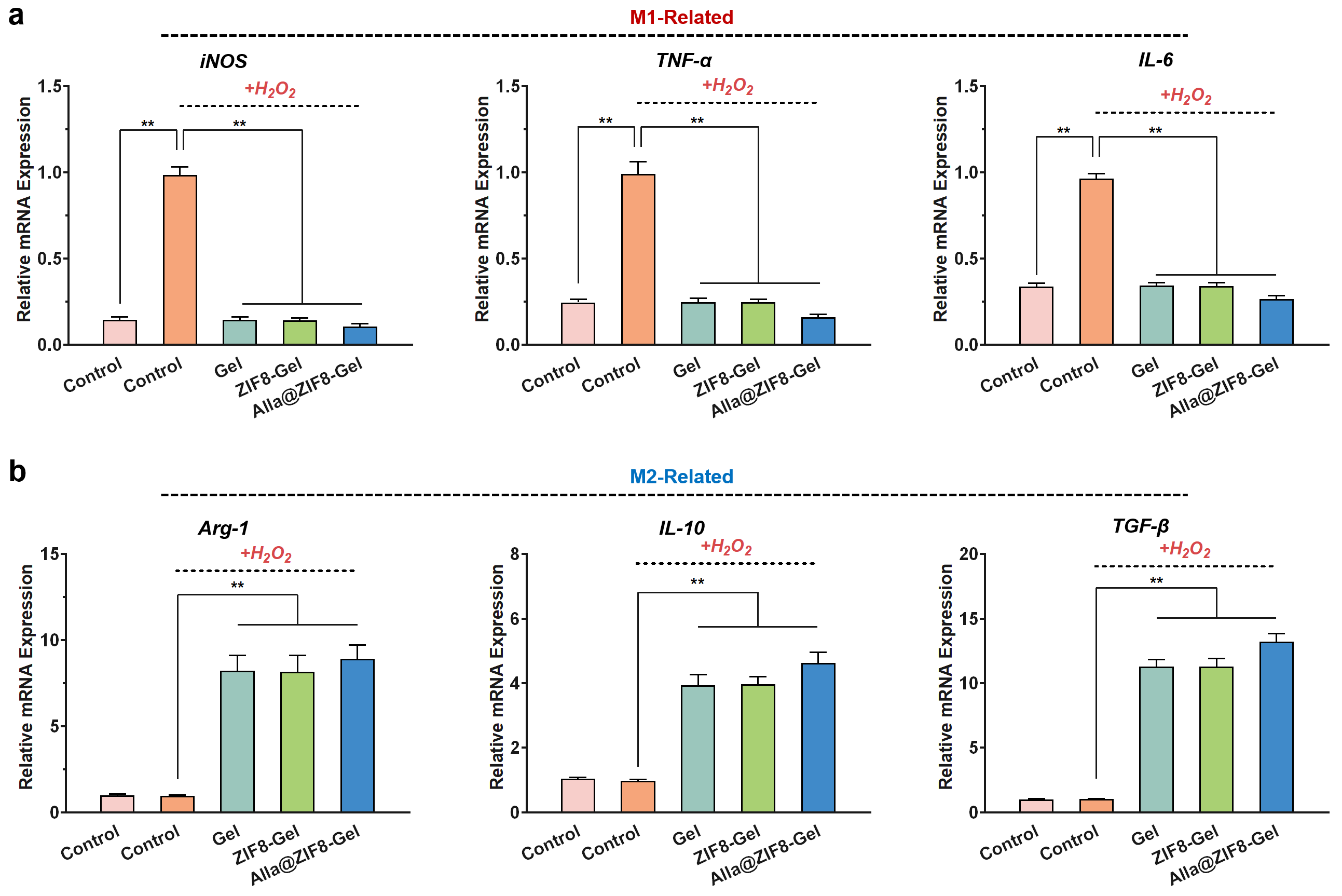


**Figure S10.** (a) Gene expression level of M1-related cytokines iNOS, TNF-α and IL-6 in RAW 264.7 cells after corresponding treatment. (b) Gene expression level of M2-related cytokines Arg-1, IL-10 and TGF-β in RAW 264.7 cells after corresponding treatment. Data are shown as the mean ± SD, *p<0.05 and **p<0.01. Statistical analysis between groups was conducted using One-way ANOVA.


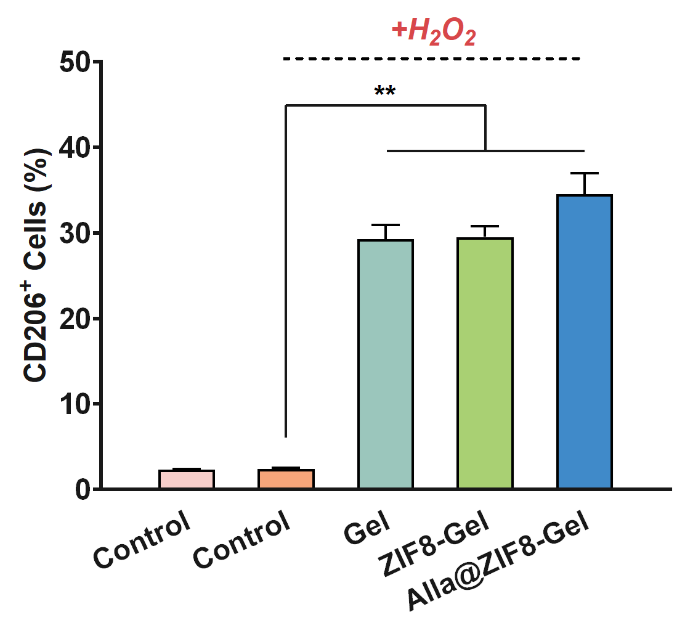


**Figure S11.** Quantitative analysis of CD206 positive RAW 264.7 cell percentage detected by flow cytometry in each group. Data are shown as the mean ± SD, *p<0.05 and **p<0.01. Statistical analysis between groups was conducted using One-way ANOVA.


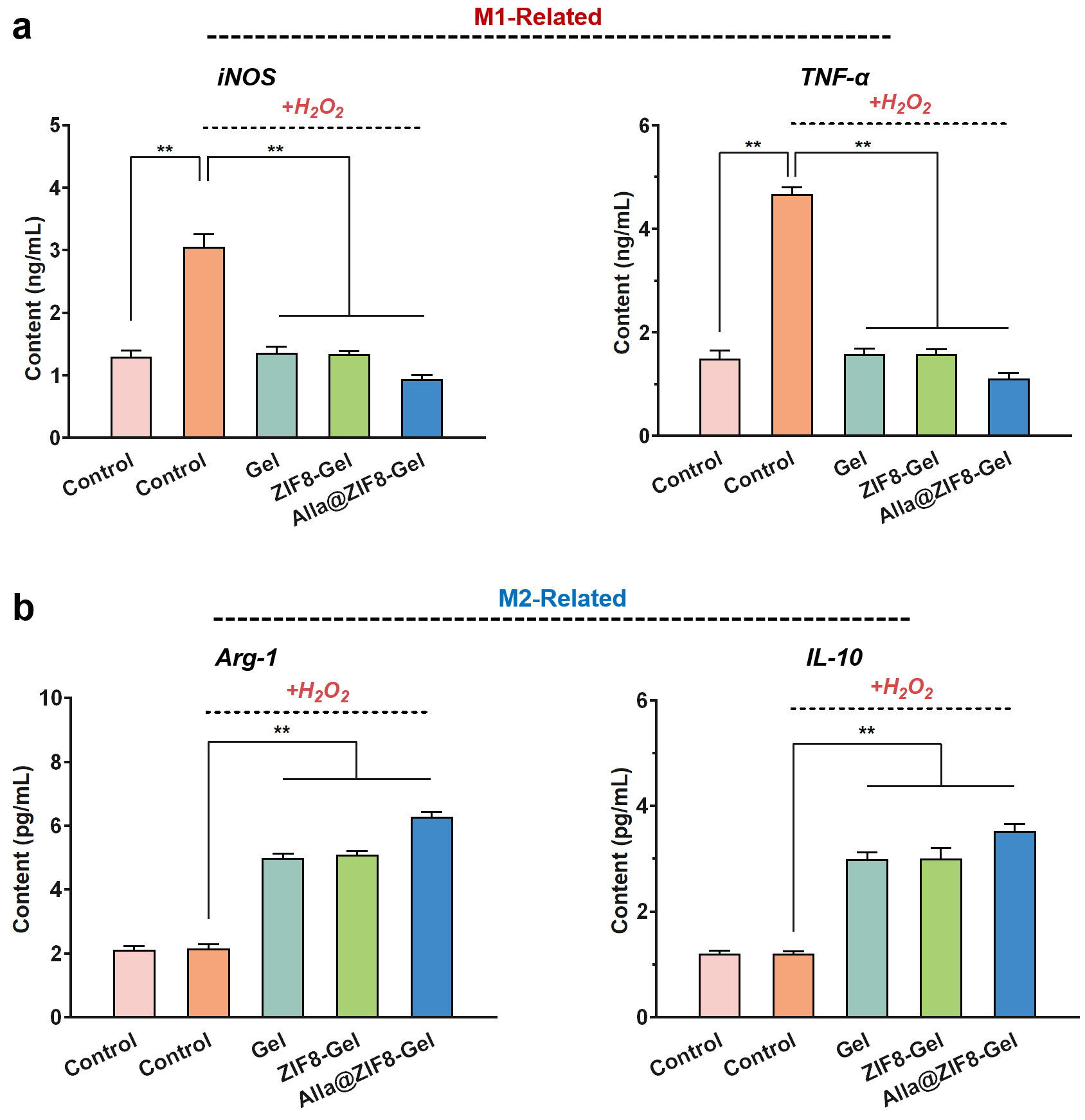


**Figure S12.** (a) The secretion of M1-related inflammatory factors (iNOS and TNF-α) from RAW 264.7 cells after corresponding treatment detected by ELISA. (b) The secretion of M2-related inflammatory factors (Arg-1 and IL-10) from RAW 264.7 cells after corresponding treatment detected by ELISA. Data are shown as the mean ± SD, *p<0.05 and **p<0.01. Statistical analysis between groups was conducted using One-way ANOVA.


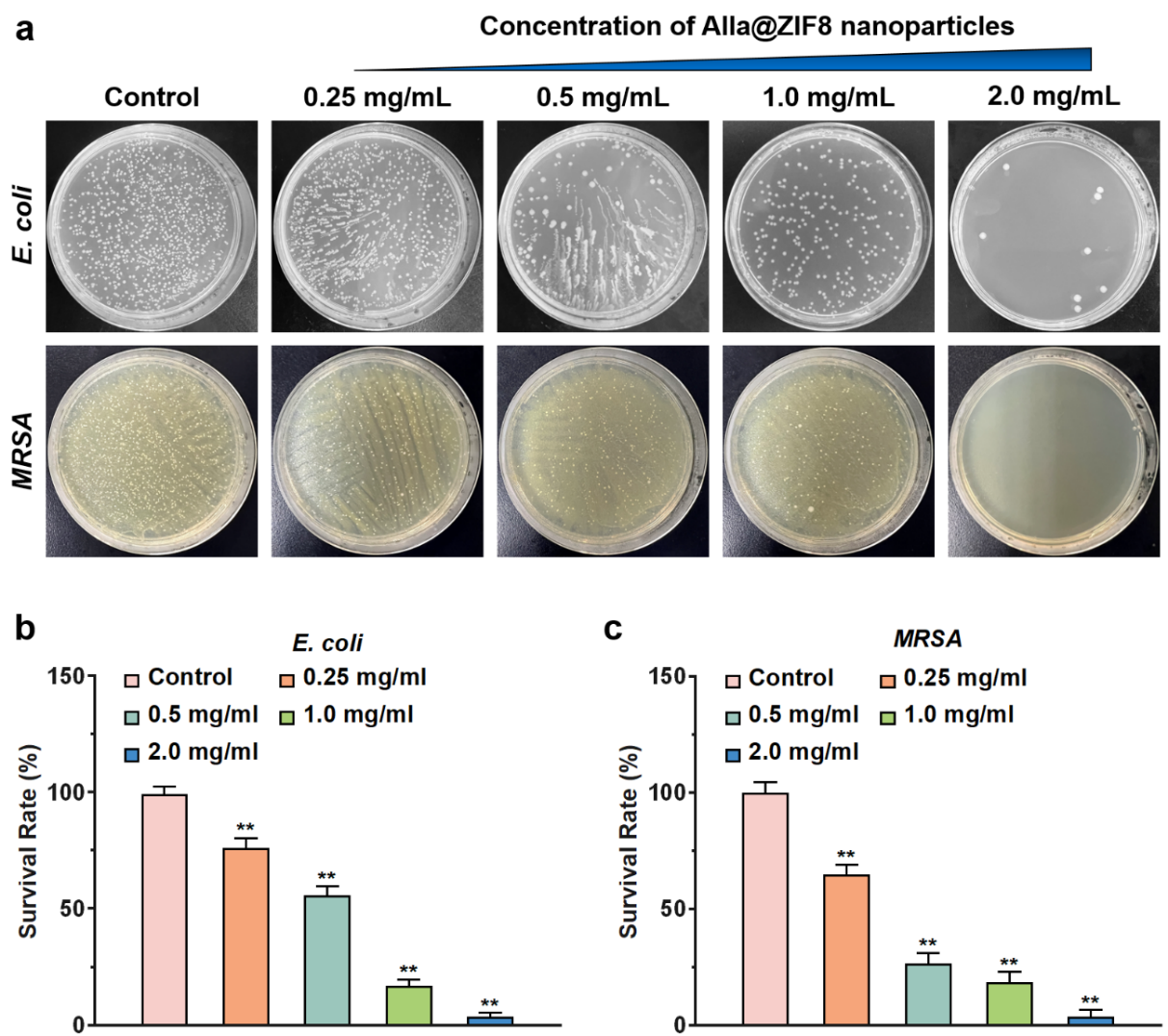


**Figure S13.** (a) Representative photographs of *E. coli* and *MRSA* CFU in Alla@ZIF8-Gel group with different content of Alla@ZIF8 nanoparticles. (b) Quantitative analysis of survival rate of *E. coli* after treatment with Alla@ZIF8-Gel with different content of Alla@ZIF8 nanoparticles. (c) Quantitative analysis of survival rate of *MRSA* after treatment with Alla@ZIF8-Gel with different content of Alla@ZIF8 nanoparticles. Data are shown as the mean ± SD, *p<0.05 and **p<0.01. Statistical analysis between groups was conducted using One-way ANOVA.


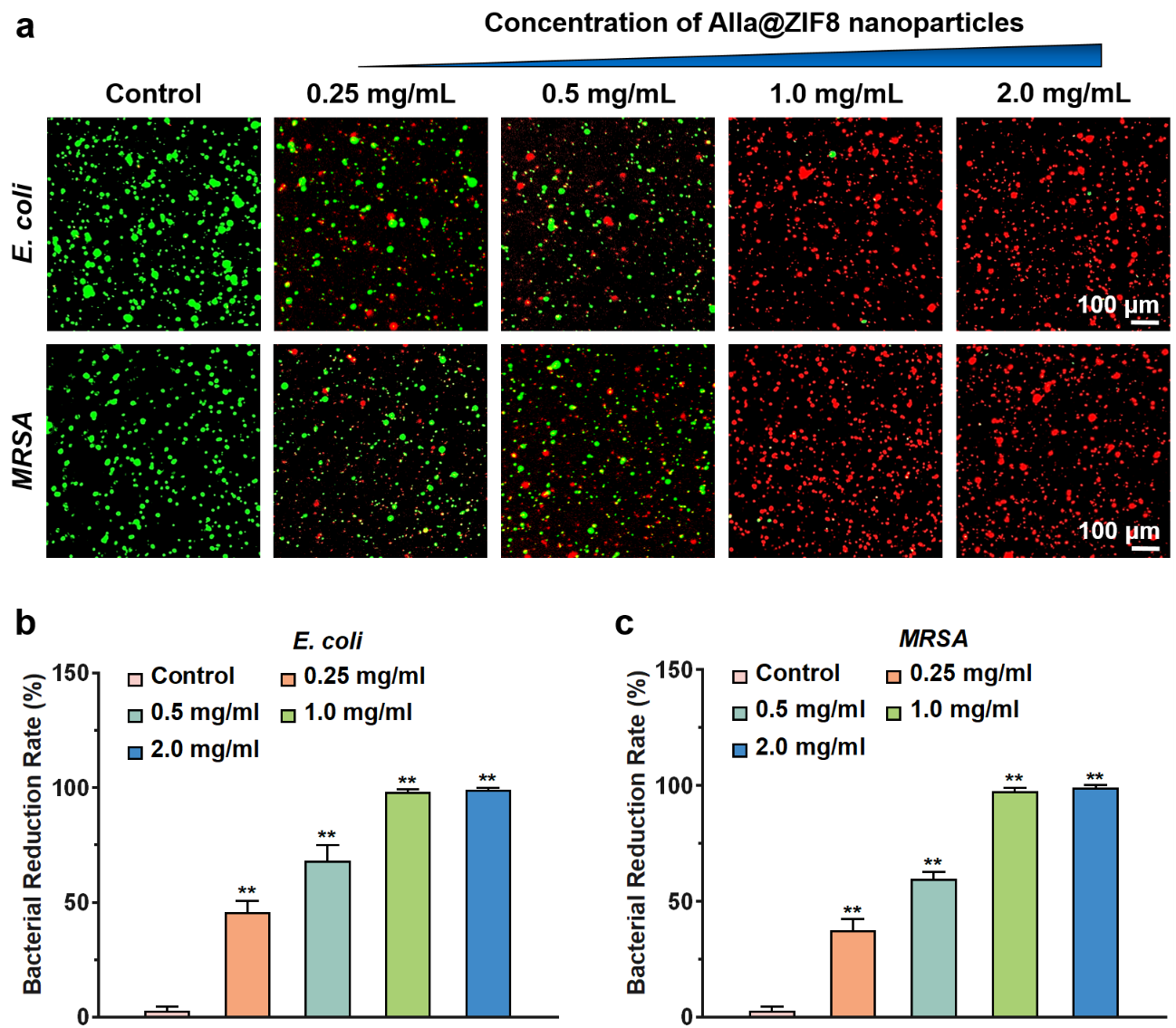


**Figure S14.** (a) Representative fluorescence images of SYTO-9/PI staining of *E. coli* and *MRSA* in Alla@ZIF8-Gel group with different content of Alla@ZIF8 nanoparticles. (b) Quantitative analysis of bacterial reduction rate of *E. coli* after treatment with Alla@ZIF8-Gel with different content of Alla@ZIF8 nanoparticles. (c) Quantitative analysis of bacterial reduction rate of *MRSA* after treatment with Alla@ZIF8-Gel with different content of Alla@ZIF8 nanoparticles. Data are shown as the mean ± SD, *p<0.05 and **p<0.01. Statistical analysis between groups was conducted using One-way ANOVA.


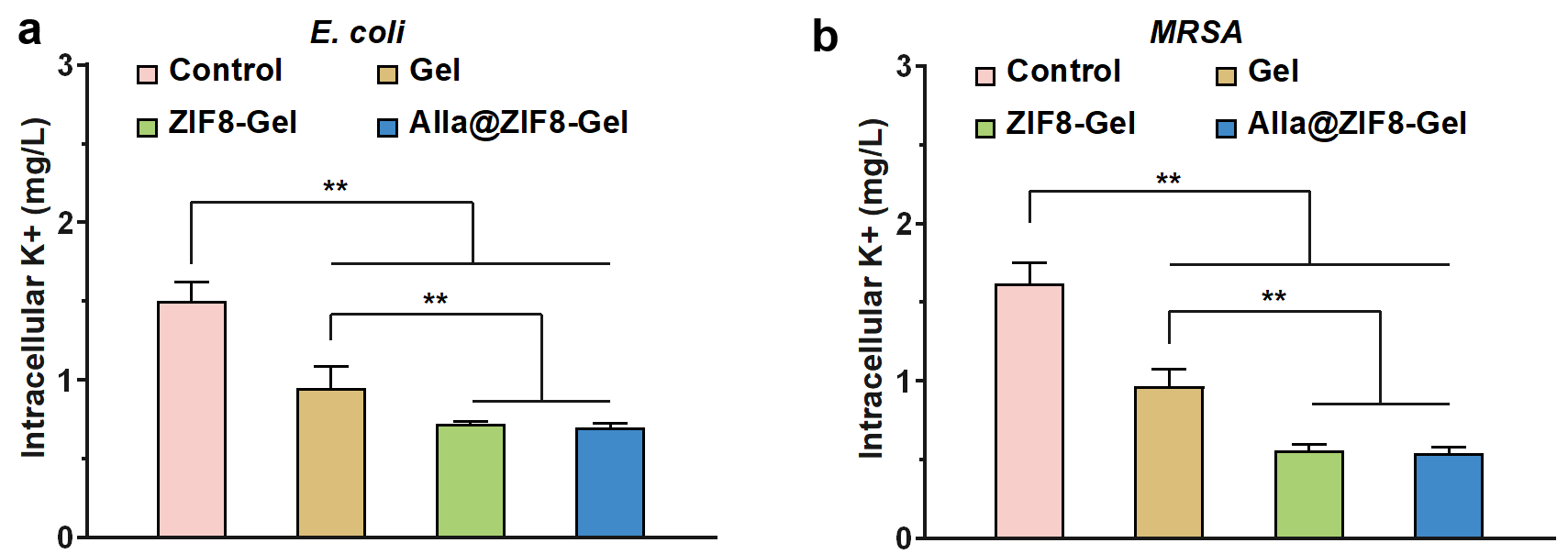


**Figure S15.** (a) Quantitative analysis of intracellular potassium ion levels in *E. coli* after treated with Alla@ZIF8-Gel. (b) Quantitative analysis of intracellular potassium ion levels in *MRSA* after treated with Alla@ZIF8-Gel. Data are shown as the mean ± SD, *p<0.05 and **p<0.01. Statistical analysis between groups was conducted using One-way ANOVA.


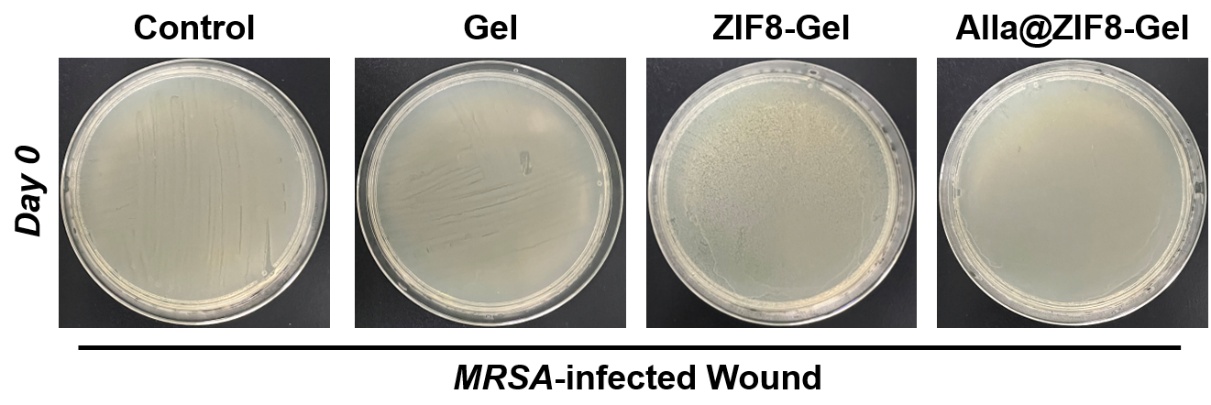


**Figure S16.** Representative photographs of *MRSA* infection in rat wounds confirmed by standard agar solid plate coating method.


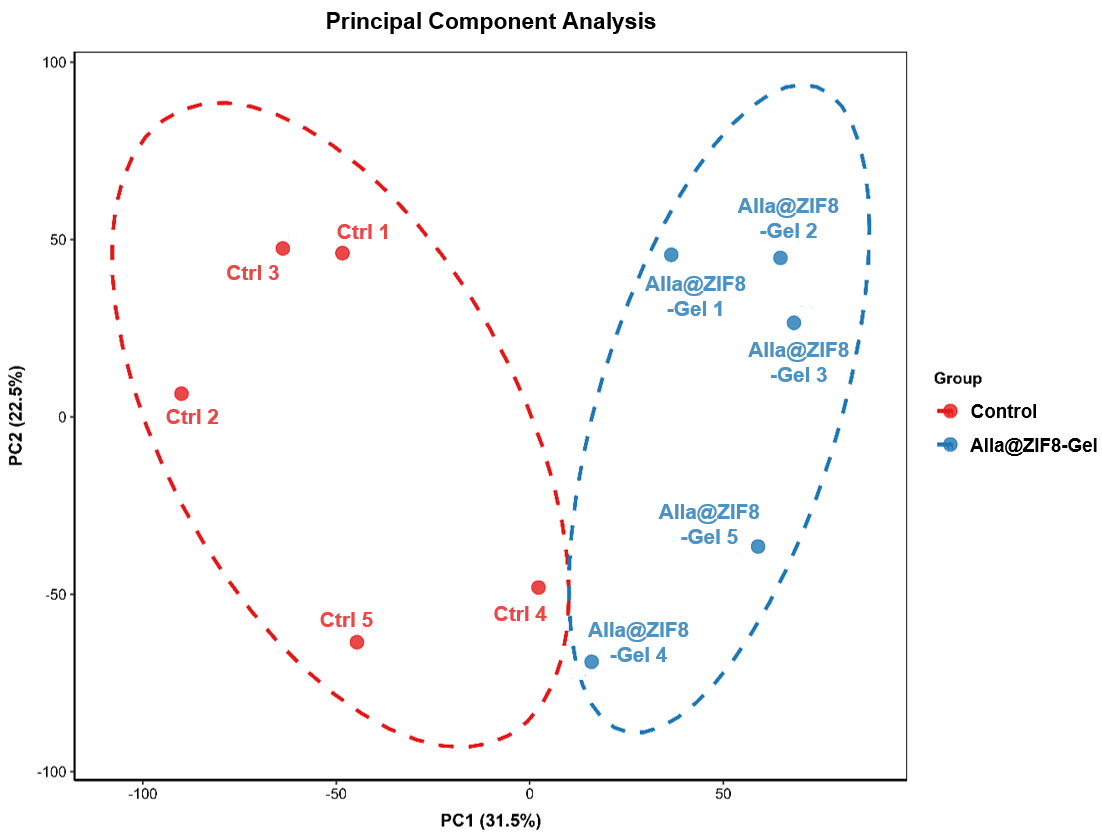


**Figure S17.** Principal component analysis (PCA) between the wound tissues of the Control group and the Alla@ZIF8-Gel group.


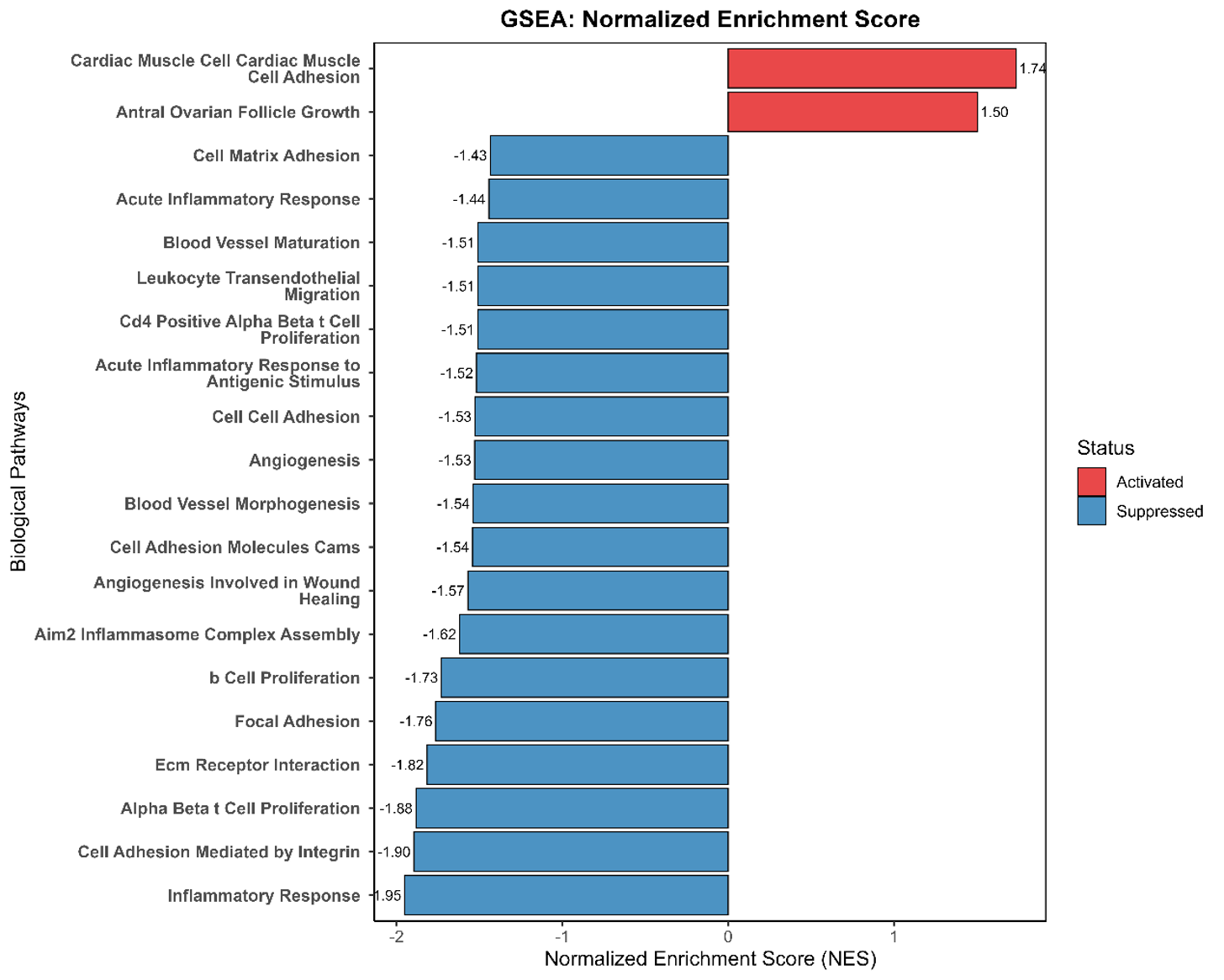


**Figure S18.** GSEA standardized enrichment score bar chart of DEGs between the Control and Alla@ZIF8-Gel group.


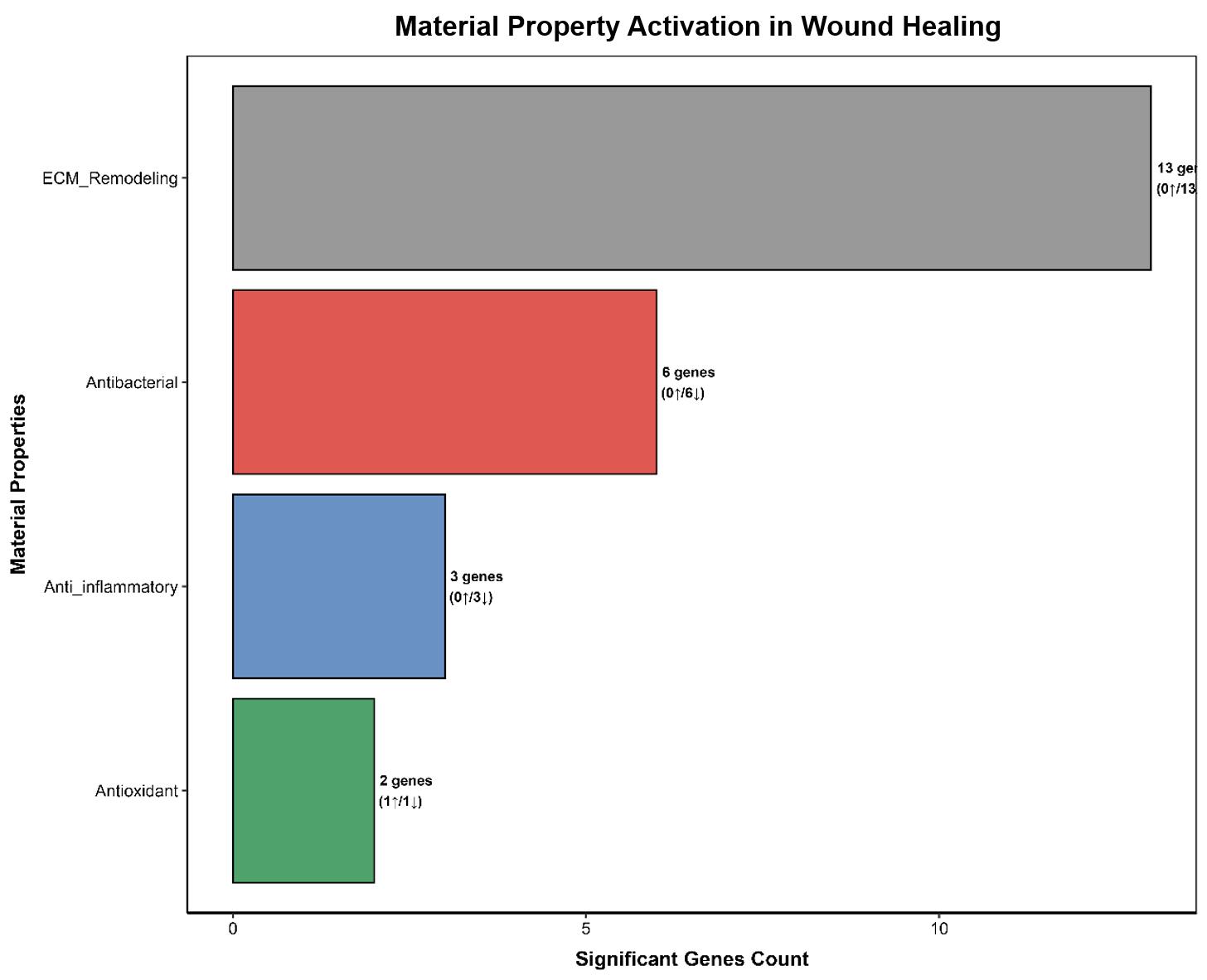


**Figure S19.** Activation analysis diagram of material property genes related to wound repair in transcriptome sequencing.


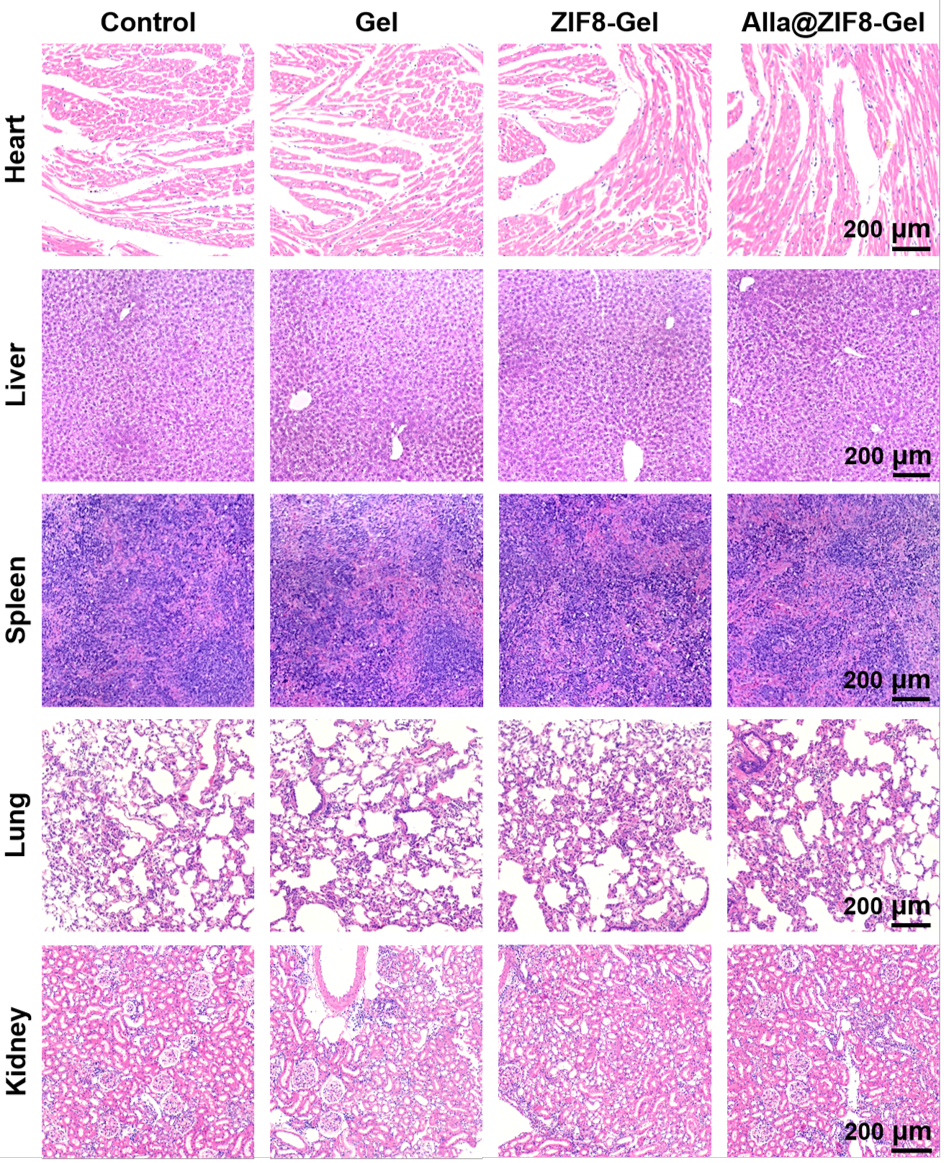


**Figure S20.** Representative photographs of HE staining of the major organs of rats in each group.


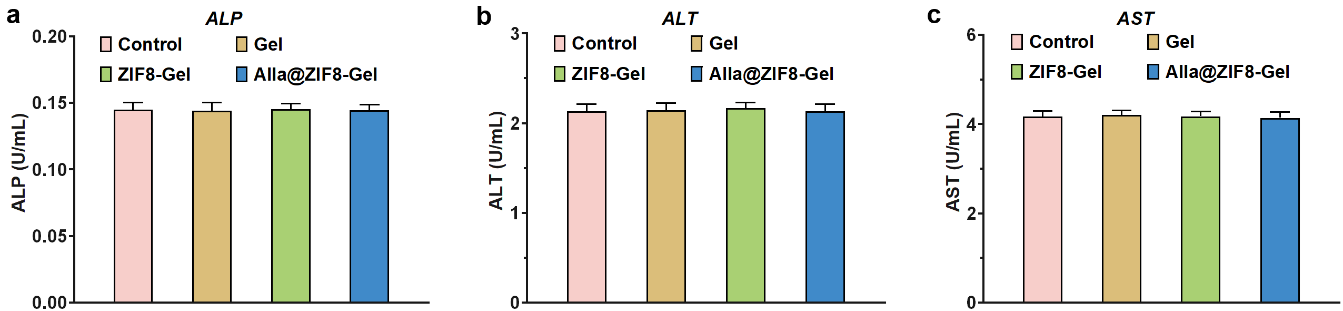


**Figure S21.** Quantitative analysis of alkaline phosphatase (ALP), alanine aminotransferase (ALT), and aspartate aminotransferase (AST) levels in peripheral blood of rats in each group. Data are shown as the mean ± SD, *p<0.05 and **p<0.01.

**Table S1.** Feed composition of the RMQCC hydrogels.

| **Sample** | **1% CMCS (g)** | **GMA (mL)** | **GTMAC (mL)** | **Res (g)** |
| --- | --- | --- | --- | --- |
| ***RMQCC_0_*** | 4 | 4 | 0 | 1 |
| ***RMQCC_2_*** | 4 | 4 | 2 | 1 |
| ***RMQCC_4_*** | 4 | 4 | 4 | 1 |
| ***RMQCC_8_*** | 4 | 4 | 8 | 1 |

**Table S2.** qRT-PCR primers

| **Gene** | **Forward primer (5’-3’)** | **Reverse primer (5’-3’)** |
| --- | --- | --- |
| ***iNOS*** | GTTCTCAGCCCAACAATACAAGA | GTGGACGGGTCGATGTCAC |
| ***TNF-α*** | CGAGTGACAAGCCTGTAGCC | ACAAGGTACAACCCATCGGC |
| ***IL-6*** | CTTCCATCCAGTTGCCTTCT | CTCCGACTTGTGAAGTGGTATAG |
| ***Arg-1*** | CTCCAAGCCAAAGTCCTTAGAG | GGAGCTGTCATTAGGGACATCA |
| ***IL-10*** | GCTGTCATCGATTTCTCCCCT | AGATGTCAAACTCATTCATGGCC |
| ***TGF-β*** | CTGCTGACCCCCACTGATAC | CTGTATTCCGTCTCCTTGGTTC |
